# The IL-17 pathway mediated by m6A-modified lncRNA H19: a new mechanism for Jianpi Qingre Tongluo Prescription in repressing inflammation and improving lipid metabolism in gout arthritis

**DOI:** 10.1186/s13020-026-01379-z

**Published:** 2026-03-18

**Authors:** Xianheng Zhang, Jian Liu, Xiaolu Chen, Xiang Ding, Shengfeng Liu, Xueni Cheng, Dahai Fang

**Affiliations:** 1 The First Affiliated Hospital of Anhui University of Chinese Medicine, Hefei, 230031 Anhui China; 2 Anhui Provincial Key Laboratory for Applied Basic and Clinical, Translational Research On Rheumatologic Diseases in Traditional Chinese Medicine, Hefei, 230009 Anhui China; 3 Anhui University of Chinese Medicine, Hefei, 230012 Anhui China

**Keywords:** Gouty arthritis, Jianpi Qingre Tongluo Prescription, Inflammation, Lipid metabolism, N6-methyladenosine modification, LncRNA H19, IL-17 pathway

## Abstract

**Background:**

Jianpi Qingre Tongluo Prescription [also named Huangqin Qingrechubi Capsule (HQC)] is an empirical prescription for the treatment of gouty arthritis (GA) with excellent clinical efficacy. Mechanistically, HQC suppresses inflammation and lipid metabolism imbalance in GA by regulating long non-coding RNA H19 (lncRNA H19). Nevertheless, the detailed mechanism requires further investigation.

**Purpose:**

This study further explored the mechanism of HQC in suppressing inflammation and improving lipid metabolism via lncRNA H19 in GA.

**Methods:**

A rat model of GA was established to analyze the effects of HQC on joint injury, inflammation, and lipid metabolism in GA. Subsequently, network pharmacology was employed to identify the key pathway involved in the effects of HQC on inflammation and lipid metabolism in GA. Based on clinical and animal experimental observations, a co-culture model of GA-peripheral blood mononuclear cells and GA-fibroblast-like synoviocytes was constructed to validate the mechanism of HQC in regulating GA-related inflammation and lipid metabolism from the perspective of N6-methyladenosine (m6A) modification of lncRNA H19.

**Results:**

HQC alleviated joint injury and improved the abnormal levels of inflammatory factors (hs-CRP, IL-4, IL-1β, and TNF-α) and lipid metabolites (TC, TG, lipoprotein, adiponectin, leptin, visfatin, and resistin) in GA rats. The IL-17 pathway was identified as an important node in HQC's effects on improving inflammation and lipid metabolism in GA. Alterations of lncRNA H19 and the IL-17 pathway were observed in GA patients and rats, which were closely correlated with inflammation and lipid metabolites. Cellular experiments revealed that high expression of lncRNA H19, attributed to ALKBH5/FTO-mediated demethylation, facilitated inflammation and lipid metabolism imbalance in GA via activating the IL-17 pathway. HQC could repress inflammation and improve lipid metabolism in GA through inhibiting the IL-17 pathway by increasing ALKBH5/FTO-mediated m6A modification of lncRNA H19; these effects might be achieved by Carthamidin.

**Conclusion:**

HQC inhibited inflammation and improved lipid metabolism in GA via inactivation of the IL-17 pathway by regulating m6A modification of lncRNA H19. Our findings further support the great potential of HQC as a candidate drug for GA treatment.

**Graphical Abstract:**

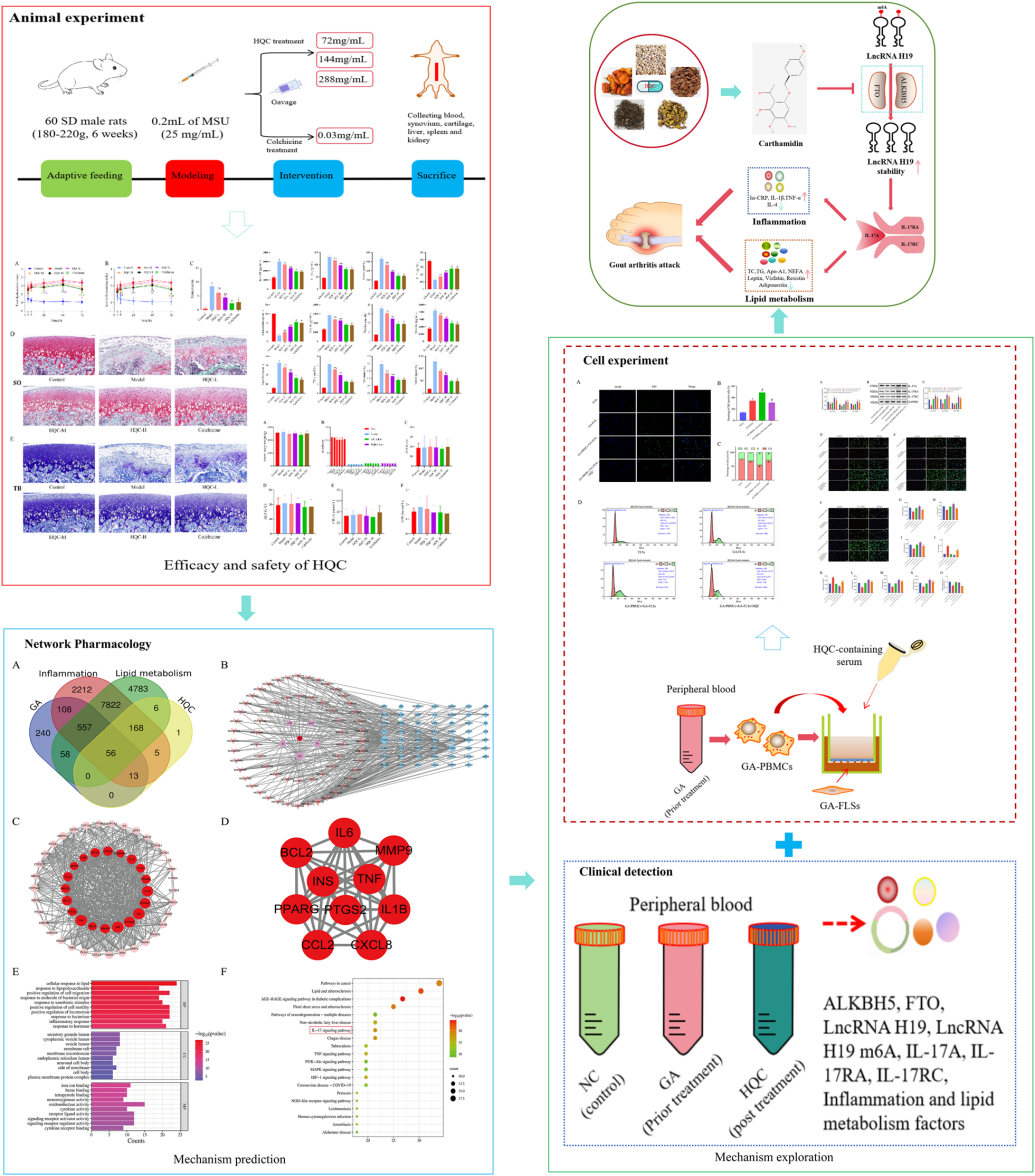

**Supplementary Information:**

The online version contains supplementary material available at 10.1186/s13020-026-01379-z.

## Introduction

Gouty arthritis (GA) is a rheumatic disease predominantly characterized by inflammatory joint pain, which is strongly associated with various risk factors (such as diet and lifestyle habits) [8]. With the rapid development of the economy, GA has become one of the major diseases affecting joint function worldwide, driven largely by its steadily increasing prevalence [20, 32]. Importantly, in addition to unbearable episodic joint pain, multiple GA-associated comorbidities (such as cardiovascular and cerebrovascular diseases and kidney damage) should also be emphasized as they pose a heavy burden on patients' quality of life and medical management [14]. It is widely recognized that joint pain and extraarticular manifestations of GA are mediated by acute and chronic inflammation. Moreover, accumulating evidence has reported that GA is strongly associated not only with inflammatory responses but also with a spectrum of metabolic diseases, ranging from abnormal uric acid metabolism to metabolic syndrome, including glucose and lipid metabolism disorders [10, 41]. Nearly half of GA patients may encounter abnormal lipid metabolism, a common comorbidity of GA [23, 24]. This comorbidity not only results in cardiovascular and cerebrovascular diseases but also amplifies GA-associated inflammatory responses, further contributing to various complications, including kidney damage and tophi [51]. For the treatment of GA, multiple clinical guidelines mainly recommend two therapeutic strategies: anti-inflammatory therapy and uric acid-lowering therapy [47]. Nevertheless, in most cases, lipid metabolism disorders in GA are not adequately controlled, which greatly affects GA treatment and prognosis.

Traditional Chinese medicine (TCM) has a long history of treating GA and has been increasingly accepted for its mode of holistic treatment and its characteristics of safety and effectiveness [6, 59]. Intriguingly, the concept of holistic treatment is also increasingly being advocated for treating many systemic diseases in modern medicine. Jianpi Qingre Tongluo Prescription, also known as Huangqin Qingrechubi Capsule (HQC), is an empirical prescription developed by the First Affiliated Hospital of Anhui University of TCM for GA treatment (Patent Number: ZL201110095718.X) and has been awarded a registration certificate for preparation in Chinese medical institutions (Approval number: Anhui Medicine System Z20200001). HQC has been applied in the clinical treatment of GA for over 20 years, showing favorable efficacy and safety [44]. HQC is composed of 5 Chinese herbs at a ratio of 10:9:5:30:10, including *Scutellaria baicalensis* Georgi (Huangqin), *Gardenia jasminoides* J. Ellis (Zhizi), *Amygdalus persica var. nucipersica* L. (Taoren), *Coix lacryma-jobi var. stenocarpa* Oliv. (Yiyiren), and *Clematis chinensis* Osbeck (Weilingxian) (the names of these plants were checked with "World Flora Online" [www.worldfloraonline.org] on August 21, 2025). These herbs were prepared into capsules through the water extraction method at the Preparation Center of the First Affiliated Hospital of Anhui University of Chinese Medicine [50]. A previous study has identified 82 compounds in HQC by ultra-performance liquid chromatography-quadrupole time-of-flight tandem mass spectrometry (UPLC-Q-TOF–MS/MS) [16]. Additionally, 6 components (baicalin, geniposide, luteolin, oleanolic acid, coixol, and amygdalin) and 12 batches were randomly sampled, demonstrating that HQC composition was stable and its quality was controllable [9, 25]. Notably, a proteomic study has shown that HQC mitigates GA-related inflammation by modulating specific proteins such as interleukin (IL)-8 and tumor necrosis factor (TNF) receptor II [36]. Additionally, a cellular study has revealed that HQC reduces microRNA (miR)-23a-3p expression to upregulate phosphatase and tensin homolog, thereby inhibiting cardiomyocyte growth and inflammatory responses [37]. Moreover, it has been unveiled that long non-coding RNA H19 (lncRNA H19 overexpression is involved in GA-related inflammation and lipid metabolism imbalance, HQC depresses these abnormalities in GA by lowering lncRNA H19 expression [53]. Nonetheless, the specific mechanism of HQC in GA requires further exploration.

N6-methyladenosine (m6A) modification is one of the most common epigenetic modifications of RNA in eukaryotes, which is important in regulating the biological effects of gene transcription [17]. m6A modification is a dynamic and reversible process co-mediated by methyltransferases ("writers",METTL3, ZCCHC4, WTAP, and RBM15), demethylases ("erasers"; FTO and ALKBH5), and methylated reading protein ("readers"; YTHDF1, IGF2BP2, YTHDC1, and HNRNPG) [26]. Notably, accumulating studies have indicated that m6A modification affects the pathophysiological effects of lncRNAs by regulating their stability or direct function [5, 54]. For example, through its key biological role in regulating the function of lncRNAs, m6A modification is implicated in the osteogenic differentiation of stem cells and the progression and treatment of various tumors [35, 45]. Furthermore, recent studies have demonstrated that the dynamic regulation of m6A modification on lncRNA H19 exerts a dual pathogenic and palliative role in the progression of many diseases (including glioma, atherosclerosis, and cerebral ischemia–reperfusion injury), providing novel insights into the treatment of these diseases [40, 48, 49]. However, whether lncRNA H19 is regulated by m6A modification and further mediates GA pathogenesis needs to be investigated.

In this context, this study further probed the mechanism of HQC in repressing inflammation and improving lipid metabolism in GA via lncRNA H19. Specifically, HQC's effects on joint injury, inflammation, and lipid metabolism in GA were first assessed in a monosodium urate (MSU)-induced GA rat model. Subsequently, network pharmacology was used to screen the key pathway involved in HQC's effect on inflammation and lipid metabolism in GA. Finally, GA-peripheral blood mononuclear cells (PBMCs) and GA-fibroblast-like synoviocytes (FLSs) were co-cultured to analyze the mechanism of HQC in affecting inflammation and lipid metabolism in GA via m6A-modified lncRNA H19.

## Materials and methods

### Participants and clinical sample acquisition

This study included 50 GA patients hospitalized in the Rheumatology and Immunology Department of the First Affiliated Hospital of Anhui University of TCM from September 2023 to December 2024. Additionally, 20 sex- and age-matched normal individuals who underwent health check-ups in the physical examination center of the hospital during the same period were included as controls. All GA patients met the 2015 international classification criteria of gout [30], and were free from comorbid infections, tumors, or other rheumatic immune diseases. GA patients were treated with HQC (1.2 g,oral administration; tid) throughout their hospitalization. The peripheral blood of GA patients and normal individuals was extracted for the separation of PBMCs using the Ficoll density gradient method. In addition, the clinical data of all participants were collected, including sex, age, course of disease, body mass index (BMI), erythrocyte sedimentation rate (ESR), hypersensitive C-reactive protein (hs-CRP), total cholesterol (TC), triglyceride (TG), high-density lipoprotein cholesterol (HDL-C), low-density lipoprotein cholesterol (LDL-C), lipoprotein α (LPα), apolipoprotein A1 (Apo-A1), apolipoprotein B (Apo-B), blood uric acid (BUA), visual analog scale (VAS) scores, aspartate aminotransferase (AST), alanine aminotransferase (ALT), blood urea nitrogen (BUN), and creatinine (CREA). This study followed the guidelines of the Declaration of Helsinki and Tokyo for humans and was approved by the Medical Ethics Committee of the First Affiliated Hospital of Anhui University of TCM (approval number: 2023AH-52). All participants signed informed consent forms.

### Cell co-culture

Ankle-derived human primary FLSs (RAB-iCell-s004) and GA-FLSs (HUM-iCell-s036) were obtained from iCell Bioscience (Shanghai, China) and cultured in Dulbecco's Modified Eagle Medium (encompassing 10% fetal bovine serum, 100 IU/mL penicillin, and 100 IU/mL streptomycin). After cells reached 80–90% confluence, they were trypsinized and passaged. Afterward, the cell co-culture model was constructed utilizing a Transwell chamber. In short, GA-FLSs were placed in the lower chamber of the Transwell system. After GA-FLSs adhered to chamber surface, GA-PBMCs were seeded into the upper chamber of the Transwell system for culture in an incubator (37℃, 5% CO_2_). According to a previous study [53], the optimal ratio for the co-culture of GA-PBMCs and GA-FLSs is 3:1 (3 × 10^6^: 1 × 10^6^), and the optimal duration is 48 h. When cells reached approximately 80% confluence, GA-FLSs were harvested for subsequent experiments.

### Cell transfection

Short-hairpin RNAs targeting lncRNA H19 (sh-lncRNA H19) and ALKBH5 (sh-ALKBH5), as well as their negative control (sh-NC) were constructed using lentiviral vectors. Meanwhile, overexpression vectors of IL-17A (OE-IL-17A), ALKBH5 (OE-ALKBH5), and FTO (OE-FTO), as well as empty vectors (OE-NC), were constructed (Table S1). Vectors and shRNAs were transfected into the co-cultured GA-FLSs, and the transfection efficiency was detected after 48 h of transfection. Specifically, the process was divided into the following steps. (1) GA-FLSs in the logarithmic growth phase were trypsinized, resuspended in complete medium, and seeded (2 × 10^5^ cells/well) into 6-well plates. Next, the plates were placed in the incubator overnight to allow the cells to fully adhere to the plate and reach the logarithmic growth phase. (2) The virus mixture was prepared. To achieve high infection efficiency, gradient pre-experiments were designed for the multiplicity of infection (MOI) (MOI = 5, 10, 20). The lentivirus volume required for achieving high transfection efficiency was calculated under a specific MOI. The virus stock was diluted in complete medium containing Polybrene (a final concentration of 4 − 8 µg/mL) and gently mixed. (3) Subsequently, the plates were collected from the incubator. After the medium was discarded, the virus mixture was added, and the plates were placed in the incubator overnight. After that, the medium containing the virus was renewed with fresh complete medium. (4) After 48 h of culture, the infection efficiency was preliminarily observed under a fluorescence microscope. (5) Cells were subcultured at a 1:3 ratio, and the medium was renewed with complete medium containing Puromycin for purification to obtain stably transfected cell lines.

### Animals and treatment

A total of 60 specific pathogen-free (SPF)-grade Sprague Dawley (SD) male rats (180 − 220 g; 6 weeks) were purchased from Liaoning Changsheng Biotechnology Co., Ltd. (Liaoning, China). Rats were raised in the Animal Experimental Center of the First Affiliated Hospital of Anhui University of TCM with a 12 h/12 h light–dark cycle, with ad libitum access to food and water. Following a 1-week acclimation, all rats were randomized into the control, model, HQC-low dose (HQC-L; 72 mg/mL), HQC-medium dose (HQC-M; 144 mg/mL), HQC-high dose (HQC-H; 288 mg/mL), and colchicine (0.03 mg/mL) groups, with 10 rats per group. Except for the control group, the GA model was successfully established in all rats [7]. Specifically, 0.2 mL of MSU suspension (25 mg/mL) was injected into the right ankle of the rat until the joint capsule bulged on the opposite side. Meanwhile, control rats were injected with the same volume of normal saline in the right ankle. After the model was successfully replicated, rats were gavaged with HQC (2 mL/rat) once a day for 7 days. Rats in the control and model group were given the same amount of normal saline [62]. One hour after the last gavage, rats were intraperitoneally injected with pentobarbital sodium for anesthesia, followed by the collection of abdominal aorta blood, synovium, cartilage, liver, spleen, and kidneys. This animal experiment was ratified by the Experimental Animal Ethics Committee of Anhui University of TCM (approval number: AHUCM-rats-2023020).

### Preparation of HQC-containing serum

Ten SPF-grade male SD rats were bought from the Experimental Animal Center of Anhui Medical University. HQC-containing serum was prepared as described previously [42]. This animal experiment was reviewed and approved by the Experimental Animal Ethics Committee of Anhui University of TCM (approval number: AHUCM-rats-2021022).

### Reverse transcription-quantitative polymerase chain reaction (RT-qPCR)

As previously reported [39], total RNA in the target samples was extracted using TRIzol reagents and then reverse transcribed into cDNA with the PrimeScript™ RT Kit (TaKaRa, Tokyo, Japan). After that, fluorescence quantitative PCR was performed. Primers and probes were synthesized by Sangon (Shanghai, China). Detailed primer sequences are listed in Table S1.

### Methylated RNA immunoprecipitation (MeRIP)-qPCR

The m6A modification of lncRNA H19 was tested with the EpiMark® N^6^-Methyladenosine Enrichment Kit (NEB#E1610S; New England Biolabs, Ipswich, Massachusetts, USA) as instructed in the manufacturer’s protocols. Specifically, total RNA was extracted and fragmented. Half of the RNA fragments were utilized for immunoprecipitation, and the other half was reserved as Input RNA. Protein A/G magnetic beads and anti-m6A antibodies were pre-mixed and incubated (4 °C, 2 h). Afterward, fragmented RNA was added to the solution of the above magnetic bead-antibody complex and incubated (4℃, 2 h) to bind the m6A-modified RNA fragments to the antibodies. After separation on a magnetic rack, the magnetic beads were resuspended with washing buffer and fully washed 3 − 5 times. The m6A-modified fragments were recovered by adding lysis buffer and labeled as IP RNA. Finally, lncRNA H19 expression was quantified through RT-qPCR.

### Enzyme-linked immunosorbent assay (ELISA)

Following collection of serum or cell supernatants, the levels of hs-CRP (JYM1181Hu/JYM0253Ra), IL-4 (JYM0142Hu/JYM0647Ra), IL-1β (JYM0083Hu/JYM0419Ra), TNF-α (JYM0110Hu/JYM0635Ra), adiponectin (JYM0870Hu/JYM0584Ra), leptin (JYM0737Hu/JYM0492Ra), resistin (JYM0872Hu/JYM0593Ra), visfatin (JYM1558Hu/JYM0165Ra), Apo-A1 (JYM0406Ra), IL-17A (JYM1702Hu), IL-17RA (JYM2850Hu), and IL-17RC (JYM2851Hu) were measured according to the protocol of the corresponding ELISA kits.

### Western blotting

Cells or synovial samples were added to the radioimmunoprecipitation assay (RIPA) lysis buffer. After centrifugation (15 min, 12,000 rpm), the supernatant was obtained. Protein samples were added with a fourfold protein loading buffer and water-bathed, followed by cooling, sample loading, electrophoresis, and membrane transferring. Next, the membranes were blocked with 5% skim milk powder for 2 h and successively incubated with primary antibodies (ALKBH5, FTO, IL-17A, IL-17RA, and IL-17RC) and horseradish peroxidase-labeled secondary antibodies. Finally, the membranes were developed with an electrogenerated chemiluminescence kit.

### 5-ethynyl-2’-deoxyuridine (EdU) assay

Cell proliferation was evaluated with an EdU488 cell proliferation kit (Beyotime, Shanghai, China). Images were captured using a digital slide scanner (Pannoramic MIDI; 3DHISTECH, Budapest, Hungary). The percentage of positive cells was quantified with ImageJ software.

### Flow cytometry (FCM)

Cell cycle distribution was assessed with FCM. In short, trypsinized cells were washed twice with phosphate-buffered saline (PBS) and fixed (12 h) with 75% ethanol precooled at -20℃. Cells were incubated (15 min, 4℃) with propidium iodide/RNase storage solutions (0.5 mL; 1,228,040; BD, Franklin Lakes, NJ, USA) in the dark. Cell cycle analysis was performed on a flow cytometer.

### Colorimetric analysis

The EpiQuik™ m6A RNA methylation quantification kit (Colorimetry) was employed to detect the m6A level of total RNA in the samples. In brief, 200 ng of RNA, along with negative control and diluted positive control, was placed into the designated well. After incubation and development, m6A levels were assessed by measuring the absorbance at 450 nm.

### Immunofluorescence

The protein levels of IL-17A, IL-17RA, and IL-17RC were determined with immunofluorescence [38]. Samples were first incubated with primary antibodies of IL-17A, IL-17RA, or IL-17RC. After washing, samples were incubated with secondary antibodies (goat anti-rabbit Immunoglobulin G [fluorescein isothiocyanate]; 1:400) and washed. a digital slide scanner was employed for observation and photography.

### Luciferase reporter gene assay

SRAMP software (http://www.cuilab.cn/sramp/) was utilized to predict two m6A methylation sites of lncRNA H19 (1615, 2286) (Fig. S1). Based on the predicted sites, wild-type (WT) and mutant (MUT1: mutation site 1615; MUT2: mutation site 2286) fragments of lncRNA H19 were constructed and inserted into pmirGLO vectors, respectively. These luciferase reporter plasmids were co-transfected with sh-NC or sh-ALKBH5 into the co-cultured GA-FLSs using Lipofectamine 2000. After 48 h of transfection, firefly and Renilla luciferase activities were measured utilizing the luciferase reporting assay kit (Promega, Madison, WI, USA).

### Actinomycin D assay

LncRNA H19 stability was assessed using the actinomycin D assay. The co-cultured GA-FLSs were exposed to 10 µg/mL of Actinomycin D (AbMole, Houston, Texas, USA). LncRNA H19 expression was tested with RT-qPCR at 0, 2, 4, 6, and 8 h.

### Cellular thermal shift assay (CETSA)

The co-cultured GA-FLSs were lysed with RIPA cell lysis buffer and centrifuged (12,000 × g, 15 min), with the supernatant collected. Proteins in the supernatant were equally divided into two tubes, with one tube receiving an equal volume of dimethyl sulfoxide (DMSO) and the other receiving an equal volume of Carthamidin to prepare Carthamidin at a final concentration of 300 μg/mL. Both tubes were then incubated at room temperature for 60 min. Each histone was evenly divided into 200 μL Eppendorf tubes (100 μL/tube). Samples in the DMSO and Carthamidin groups were heated at 40, 45, 50, 55, 60, 65, 70, and 80℃ for 3 min, respectively, followed by cooling at room temperature for 3 min. The protein levels of ALKBH5 and FTO were examined with Western blotting.

### Immunohistochemistry

Paraffin blocks of synovial tissues were sliced (2 − 3 μm), baked (66℃, 30 min), dewaxed, and hydrated, followed by high-pressure thermal antigen repair in citric acid solutions (pH 6.0). The slices were naturally cooled to room temperature and then incubated with 3% hydrogen peroxide to inactivate endogenous peroxidase. After discarding the excess liquid on the slices, the slices were incubated (37℃, 60 min) with primary antibodies of IL-17A, IL-17RA, and IL-17RC, followed by incubation (37℃, 30 min) with secondary antibodies. Afterward, the slices were successively stained with 3, 3-diaminobenzidine, hematoxylin, and lithium carbonate solutions and sealed, followed by microscopic observation.

### Biochemistry analysis

The serum levels of TC (A111-1-1), TG (A110-1-1), non-esterified fatty acid (NEFA; A042-1-1), ALT (C009-2-1), AST (C010-2-1), CREA (C011-2-1), and BUN (C013-2-1) were measured following the instructions of the corresponding biochemical detection kits.

### Histological analysis

The cartilage tissues of rats were obtained to prepare paraffin sections. Changes in cartilage tissues were observed through Safranin O and fast green staining and toluidine blue staining, and the degree of cartilage injury was assessed with Mankin scores [61].

The synovium and cartilage of the right ankle were carefully dissected and sectioned into blocks (1 mm × 1 mm × 1 mm). After dehydration and embedding, ultrathin sectioning and staining were performed. The ultrastructure of synovial cells and chondrocytes was observed under transmission electron microscopy (TEM) [12].

### Foot dysfunction index (FDI) and joint inflammation index (JII)

The degree of foot dysfunction was assessed through gait observation at 4, 8, 24, 48, and 72 h after modeling. Foot dysfunction was graded at 4 levels [43]: grade 0 (0 score,normal gait and uniform foot landing); grade 1 (1 score; reduced foot landing, unextended toe, and mild claudication); grade 2 (2 scores; foot flexion, toes on the ground, and obvious lameness); grade 3 (3 scores; complete foot lift and three-legged gait). If the appropriate level was ambiguous, the midpoint score was assigned. The resultant score was FDI.

In addition, joint inflammation was also evaluated at 4, 8, 24, 48, and 72 h after modeling. The specific criteria are as follows [56]: grade 0 (0 score,normal joint and no signs of inflammation); grade 1 (1 score; mild swelling and redness of joint skin, clear bone signs, and mild inflammation); grade 2 (2 scores; local joint swelling and redness, no bone signs, and moderate inflammation); grade 3 (3 scores; swelling outside the joint area, severe inflammation, and frequent lifting of the injured limb). The obtained score was JII.

### Body weight and organ index

After the rats were anesthetized, their body weight was measured using an electronic balance. Additionally, the liver, spleen, and kidneys of the rats were collected and weighed immediately. The organ index was calculated to assess the drug safety of HQC: organ index = (organ weight/body weight) × 100%.

### Bioinformatics analysis

With drug-likeness ≥ 0.15 and oral bioavailability ≥ 20% as screening criteria, the active ingredients and corresponding target genes of 5 Chinese herbs in HQC were screened using TCMSP (https://test.tcmsp-e.com/tcmsp.php) and UniProt (https://www.uniprot.org/) databases. Additionally, with "gouty arthritis", "arthritis, gouty", "abnormal lipid profile", "lipid metabolism disorder", and "inflammation" as keywords, target genes related to inflammation and lipid metabolism in GA were obtained from the disease database (Table S2). Subsequently, the aforementioned target genes were intersected, and the HQC-target gene-disease network was visualized using the Cytoscape 3.7.2 software. After the interaction score was set at ≥ 0.4, intersection genes were imported into STRING (https://string-db.org/). Next, the TSV file was exported and then imported into Cytoscape 3.7.2 software to construct the protein–protein interaction (PPI) network. Meanwhile, the intersection genes were introduced into Metascape (https://metascape.org/) for gene ontology (GO) and Kyoto encyclopedia of genes and genomes (KEGG) enrichment analysis, followed by visualization with bioinformatics (http://www.bioinformatics.com.cn/).

Next, the binding ability of the active ingredients in HQC to ALKBH5 and FTO was analyzed. Specifically, molecular docking was performed with AutoDockTools 1.5.6 as previously described [52]. The binding energy < -5 kcal/mol was regarded as strong binding.

Based on molecular docking results, molecular dynamics simulation of the protein–ligand complex was conducted using Gromacs 2022 for 100 ns [22]. For proteins, the CHARMM 36 force field parameter was utilized, and the ligand topology was constructed with the GAFF2 force field parameter. The system was maintained at a constant temperature (310 K) and pressure (1 bar), with periodic boundary conditions applied. Complex stability was assessed by analyzing root mean square deviation (RMSD), radius of gyration (Rg), root mean square fluctuation (RMSF), solvent accessible surface area (SASA), and hydrogen bonds (HBonds).

### Statistical analysis

When data conformed to normal distribution, they were presented as mean ± standard deviation, and otherwise as quartiles. For comparisons of two independent samples, normally distributed data were analyzed using the independent samples *t*-test, while non-normally distributed data were analyzed using the Wilcoxon rank sum test. For the comparison of multiple samples, variance analysis and Student–Newman–Keuls q test were utilized for normally distributed data, while the Kruskal–Wallis H rank sum test and Nemenyi test were employed for non-normally distributed data. Multiple test corrections were conducted using the Bonferroni method. For intra-group comparisons, normally distributed data were analyzed utilizing the paired *t*-test, while non-normally distributed data were analyzed with the Wilcoxon signed-rank test. Spearman correlation analysis was employed to evaluate the correlation strength of the two indicators. *P* < 0.05 was considered statistically significant. All data were processed using IBM SPSS Statistics 23.

## Results

### HQC relieves tissue injury and joint dysfunction in GA rats

**Fig. 1:**
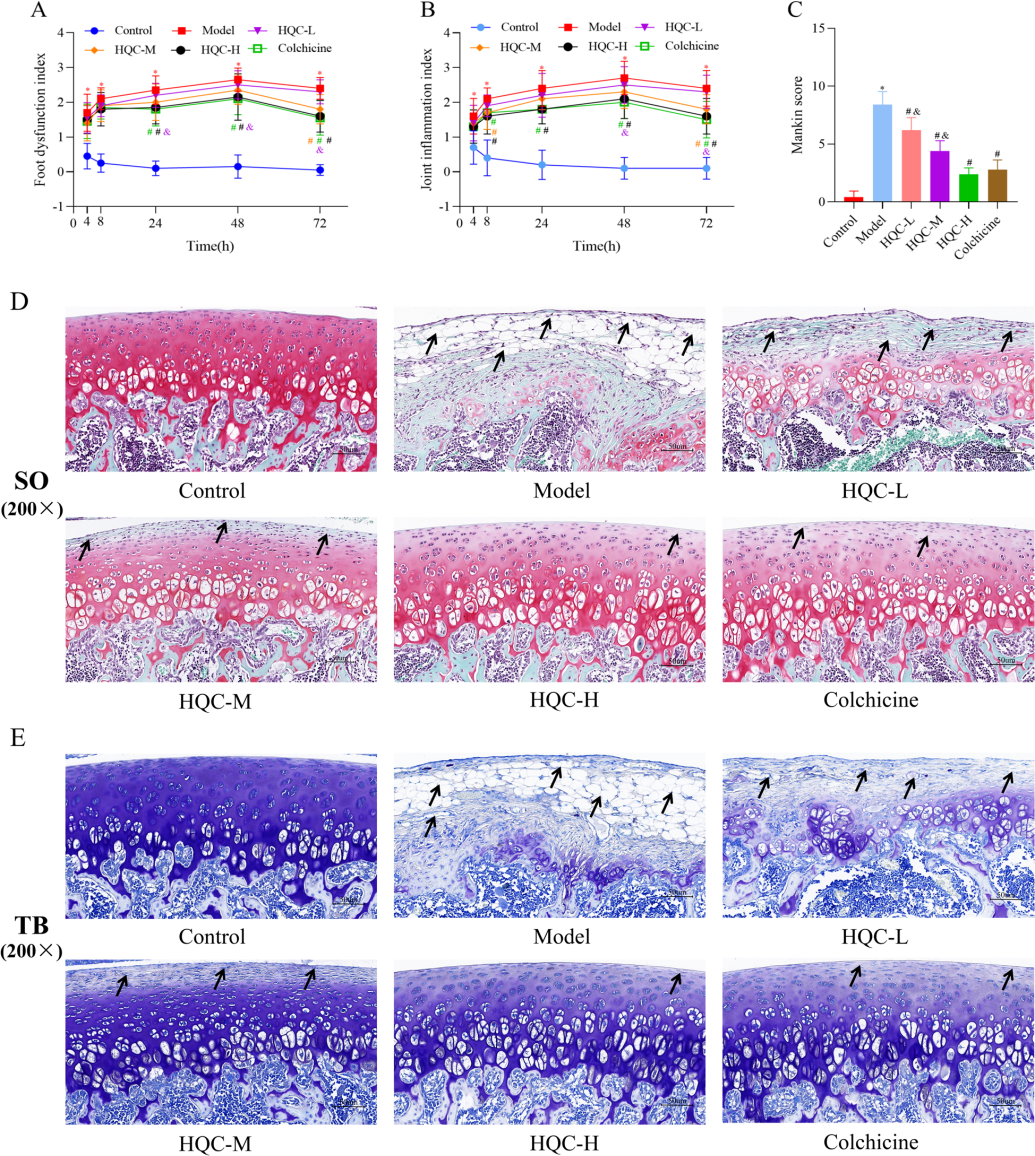
HQC improves tissue injury and joint function in GA rats. **A** FDI and **B** JII at 4, 8, 24, 48 and 72 h in each group. **C** Mankin score of rats in each group. **D** Safranin O and fast green and **E** toluidine blue staining images of cartilage tissues of rats in each group (200 × ; scale bar = 50 μm). In the Safranin O and fast green staining images: red, cartilage matrix and the cytoplasm of cartilage cells; blue, nuclei of cartilage cells; grayish green, collagen fibers and bone tissue; grayish black, nuclei. In the toluidine blue staining image: purplish red, cartilage, osteoblasts, and mast cells; blue, cell nuclei. The arrow shows the degeneration of cartilage. ^*^p < 0.05, vs. control group; ^#^*p* < 0.05, vs. model group; ^&^*p* < 0.05, vs. colchicine group

**Fig. 2:**
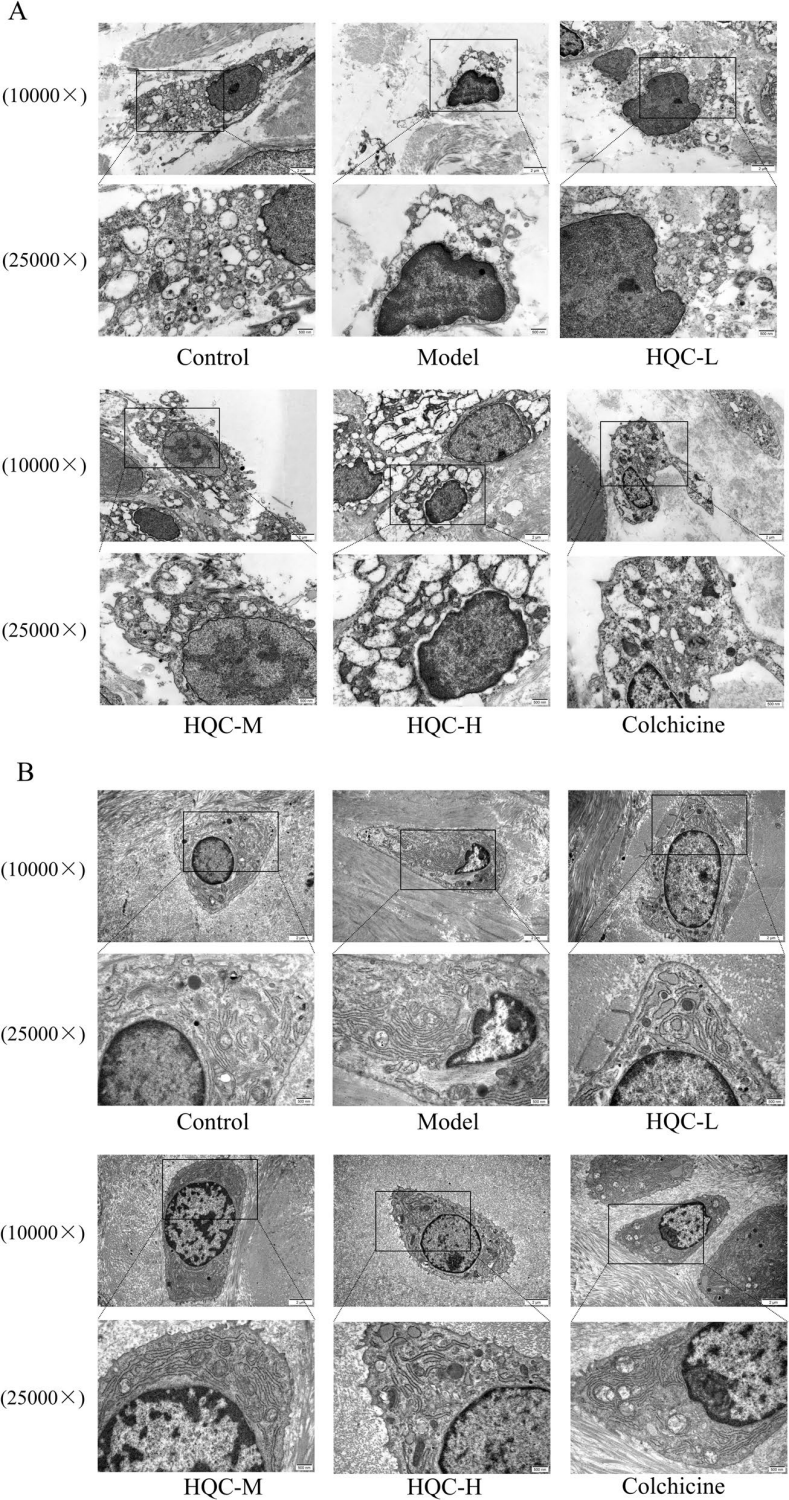
HQC improves the ultrastructure of synoviocytes and chondrocytes in GA rats. **A** Synoviocytes and **B** chondrocytes were photographed under a TEM at magnifications of 10,000 × (scale bar = 2 μm) and 25,000 × (scale bar = 500 nm)

A previous study has elucidated that HQC suppresses synovial inflammation and joint swelling in GA rats [50]. In the present study, additional observations were conducted. First, FDI and JII were calculated, showing that FDI and JII at various time points were notably higher in the model group than those in the control group,however, HQC treatment improved these two indices in GA rats to varying degrees (Fig. 1A, B). In addition, histological changes were observed through special staining and TEM. According to Safranin O and fast green staining and toluidine blue staining, the cartilage surface of the modeled rats was rough, with reduced chondrocytes and extracellular matrix, abnormal morphological changes, tidemark disruption, and evident subchondral bone thickening. Similar to colchicine, HQC improved these pathological changes in a dose-dependent manner and obviously lowered the Mankin score in GA rats (Fig. 1C–E). According to TEM results, the synovial cells and chondrocytes in the control group had intact nuclear and cell membranes, along with normal endoplasmic reticulum and mitochondria, and uniform chromatin. In contrast, synovial cells and chondrocytes in the model group showed chromatin aggregation, endoplasmic reticulum expansion and rupture, and mitochondria vacuolation, accompanied by mitochondrial cristae breakdown and disappearance. Notably, both HQC and colchicine alleviated the damage to synovial cells and chondrocytes in GA rats (Fig. 2A, B). Altogether, HQC alleviated tissue damage and joint dysfunction in GA rats.

### HQC impedes inflammation and improves lipid metabolism in GA rats

**Fig. 3:**
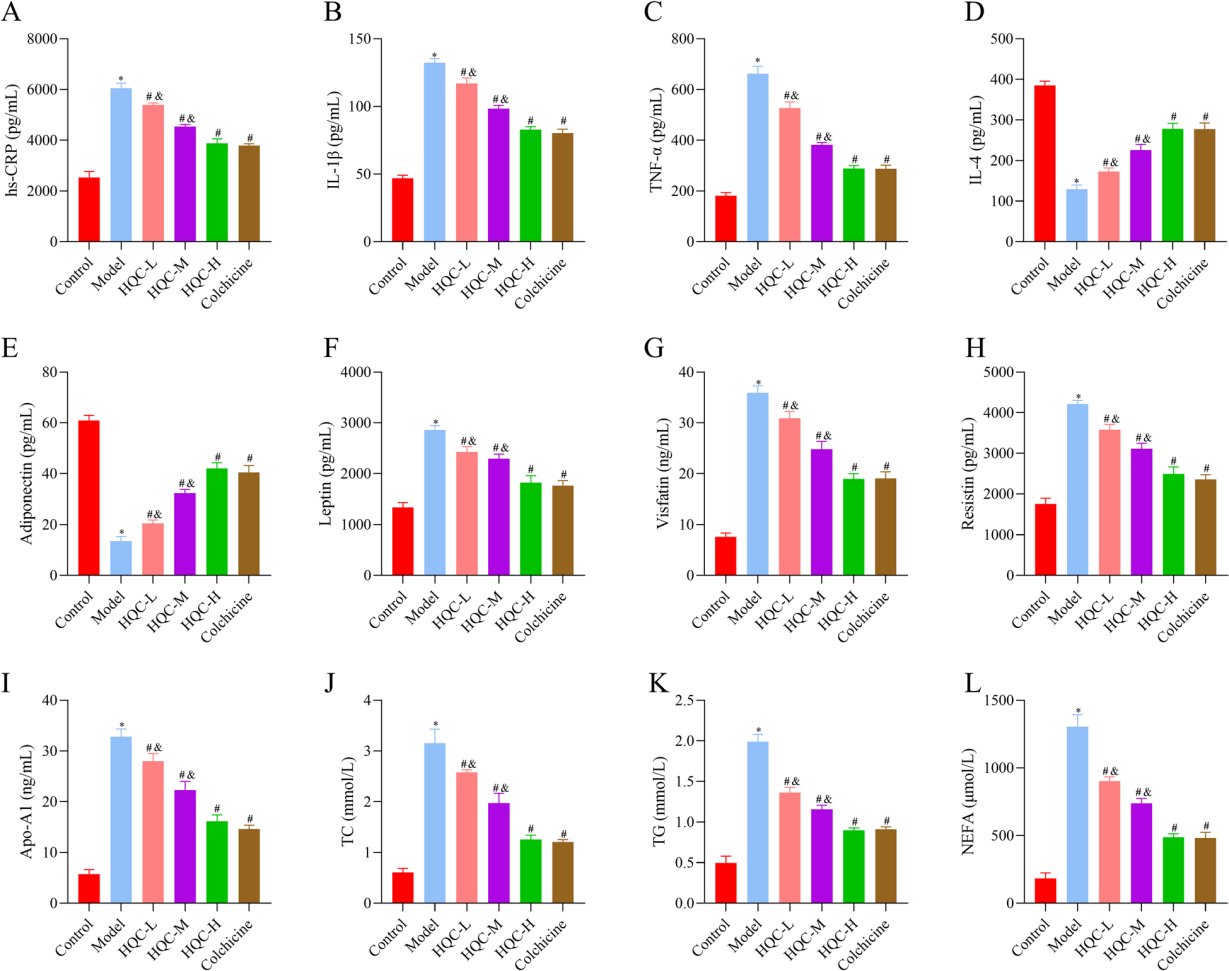
HQC alleviates inflammation and lipid metabolism imbalance in GA rats. **A**–**I** The expressions of hs-CRP, IL-1β, TNF-α, IL-4, adiponectin, leptin, visfatin, resistin, and Apo-A1 were detected by ELISA. **J**–**L** The levels of TC, TG, and NEFA were measured using the biochemical method. *hs-CRP* hypersensitive C-reactive protein, *IL-1β* interleukin-1 beta, *TNF-α* tumor necrosis factor-alpha, *IL-4* interleukin-4, *TC* total cholesterol, *TG* triglyceride, *NEFA* non-esterified fatty acid. ^*^p < 0.05, vs. control group; ^#^*p* < 0.05, vs. model group; ^&^*p* < 0.05, vs. colchicine group

**Fig. 4:**
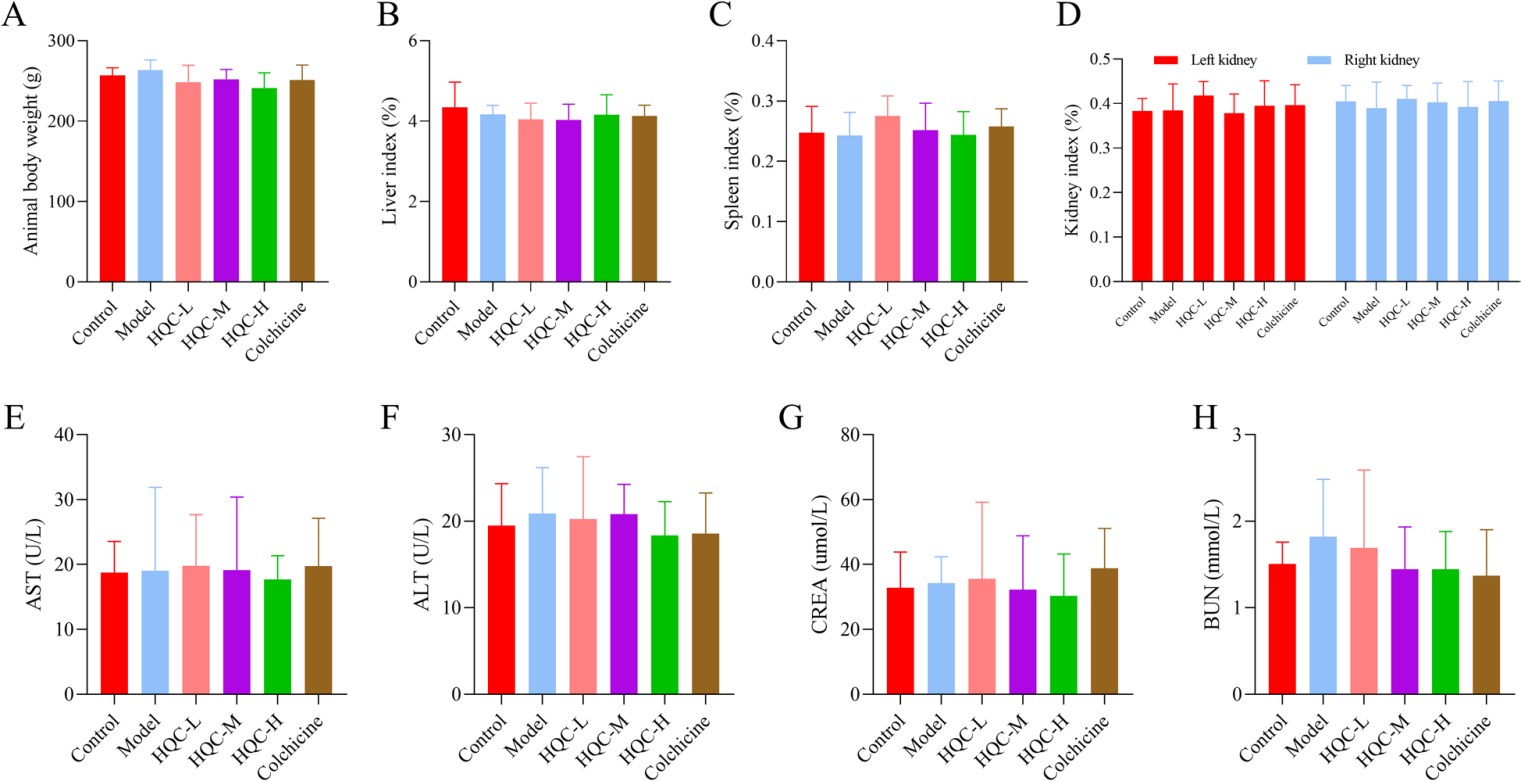
Drug safety is observed. **A** The body weight of rats in each group. **B**–**D** The liver index, spleen index, and kidney index of rats in each group were detected. **E**–**H** The levels of AST, ALT, CREA and BUN were measured using the biochemical method. *AST* aspartate aminotransferase, *ALT* alanine aminotransferase, *CREA* creatinine, *BUN* blood urea nitrogen

Further, inflammation- and lipid metabolism-related indicators were tested. As depicted in Fig. 3A–L, hs-CRP, IL-1β, TNF-α, leptin, resistin, visfatin, TC, TG, Apo-A1, and NEFA levels were prominently higher, and IL-4 and adiponectin levels were lower in the model group than those in the control group, which was nullified by HQC and colchicine. Given the importance of drug safety in clinical practice, relevant observations were also conducted. The results demonstrated insignificant differences in ALT, AST, CREA, BUN, body weight, and organ index (including liver, spleen, and kidney indexes) among all groups (Fig. 4A–H). These findings indicated that HQC attenuated inflammation and lipid metabolism disorders in GA rats with high safety.

### The IL-17 pathway is an important node for HQC in hindering inflammation and improving lipid metabolism in GA

Subsequently, network pharmacological analysis was conducted to analyze the important pathways involved in the improving effect of HQC on inflammation and lipid metabolism in GA. A total of 110 active components and 249 target genes of HQC were identified (Table S3). Meanwhile, 1032, 10941, and 13450 genes were identified to be related to GA, inflammation, and lipid metabolism, respectively. After the intersection, 56 genes (intersection genes) were determined to be involved in HQC's effect on inflammation and lipid metabolism in GA (Fig. S2A). Then, based on the intersection genes, HQC-target gene-disease and PPI networks were constructed, and the top 10 genes in terms of degree value were identified as core targets (Fig. S2B-D). After that, the intersection genes were subjected to GO and KEGG analysis. As indicated by GO analysis results, these genes were enriched in 942 biological processes (cellular response to lipid, positive regulation of cell migration, inflammatory response, etc.), 23 cellular components (secretory granule lumen, cytoplasmic vesicle lumen, membrane raft, etc.), and 71 molecular functions (receptor ligand activity, signaling receptor activator activity, cytokine activity, etc.) (Fig. S2E). The KEGG analysis results showed that these genes were enriched in 148 pathways. Based on the number of enriched genes, the top 20 entries were chosen for visualization, revealing that the IL-17 pathway ranked first (excluding pathways such as cancer and diabetes) (Fig. S2F). Of note, seven of the 10 core targets were enriched in the IL-17 pathway. All these findings suggested that the IL-17 pathway may be important in HQC-induced improvement of inflammation and lipid metabolism in GA.

**Fig. 5:**
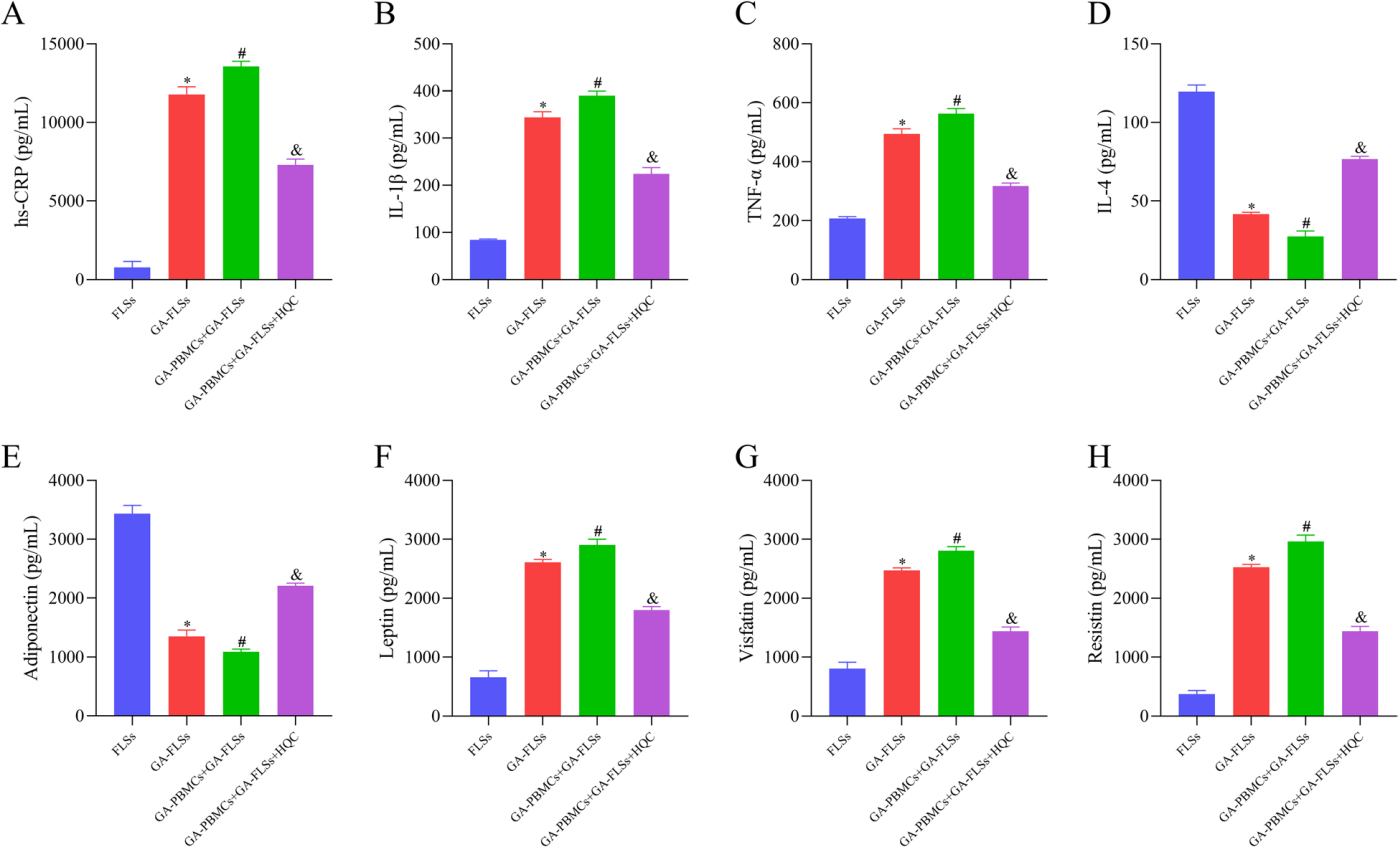
HQC improves the inflammation and lipid metabolism imbalance in the co-cultured GA-FLSs. **A**–**H** The expressions of hs-CRP, IL-1β, TNF-α, IL-4, adiponectin, leptin, visfatin, and resistin were detected by ELISA. *hs-CRP* hypersensitive C-reactive protein, *IL-1β* interleukin-1 beta, *TNF-α* tumor necrosis factor-alpha, *IL-4* interleukin-4. ^*^p < 0.05, vs. FLSs; ^#^*p* < 0.05, vs. GA-FLSs; ^&^*p* < 0.05, vs. GA-PBMCs + GA-FLSs

**Fig. 6:**
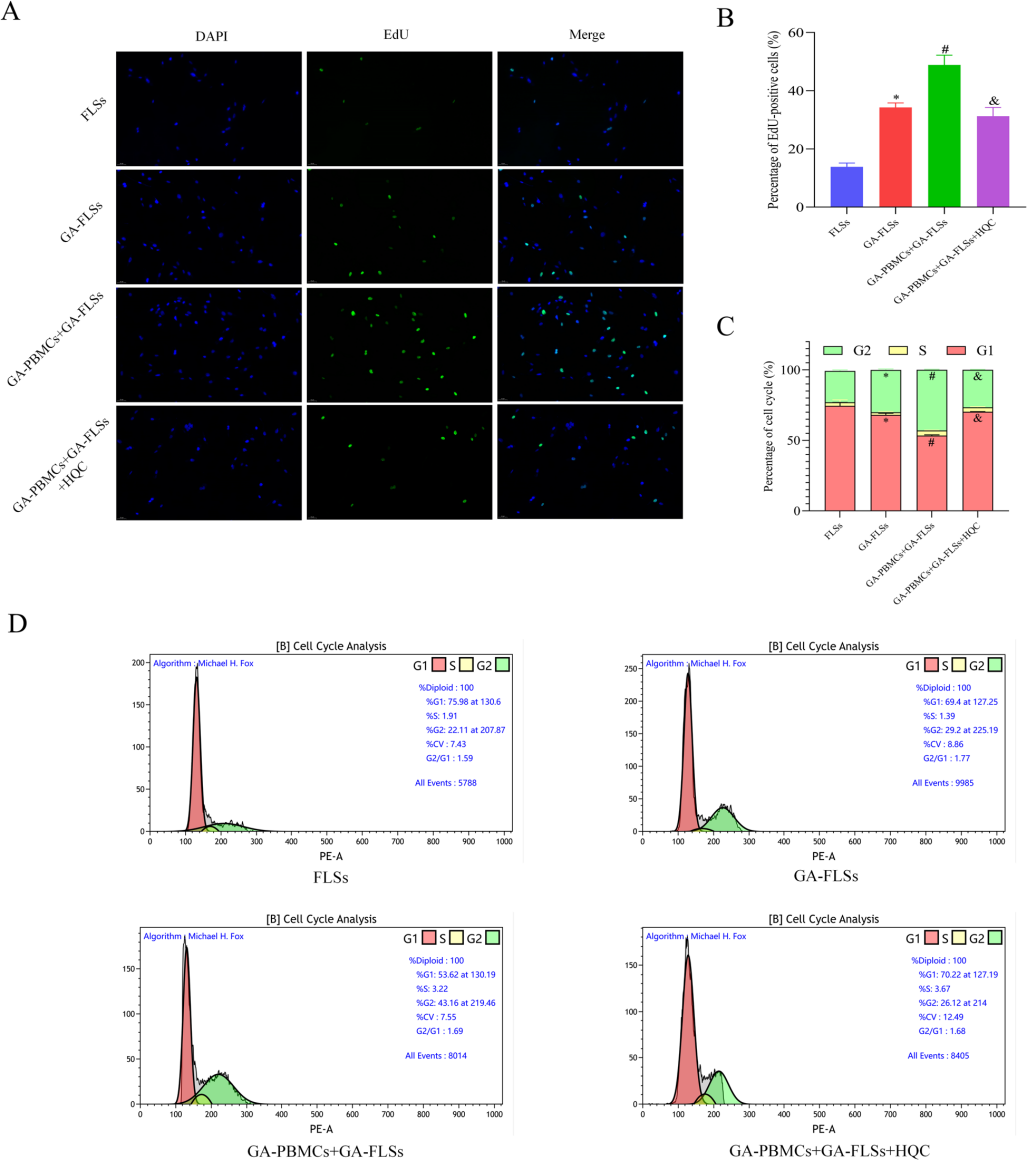
The effect of HQC on the proliferation and cell cycle distribution of the co-cultured GA-FLSs is assessed. **A**, **B** Cell proliferation was detected by the EdU assay (20 × ; scale bar = 50 μm). **C**, **D** Cell cycle distribution was detected by flow cytometry. ^*^p < 0.05, vs. FLSs; ^#^*p* < 0.05, vs. GA-FLSs; ^&^*p* < 0.05, vs. GA-PBMCs + GA-FLSs

**Fig. 7:**
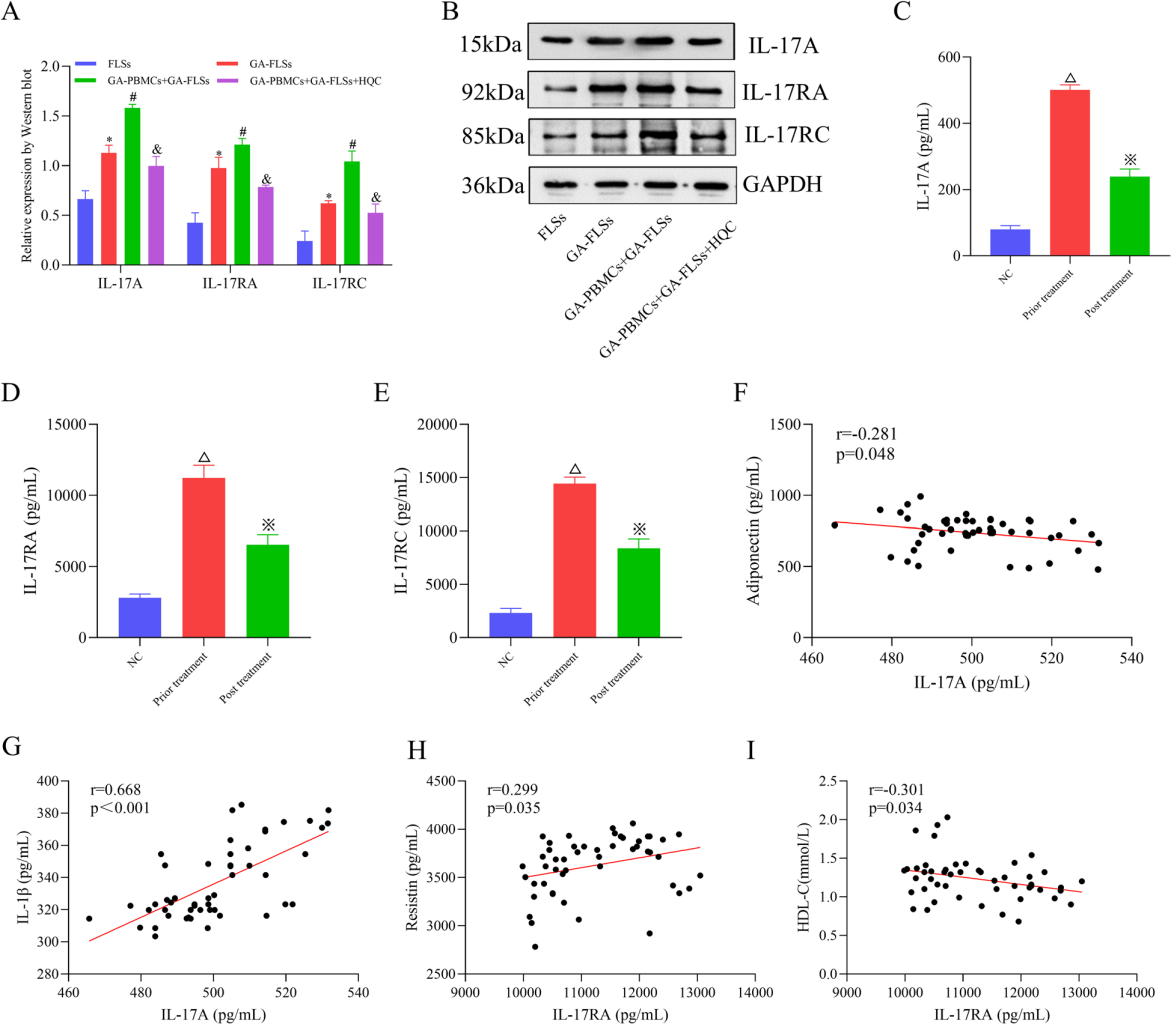
The IL-17 signaling pathway is an important node for HQC to improve GA inflammation and lipid metabolism. **A**, **B** The expression of IL-17A, IL-17RA, and IL-17RC was measured using Western blotting. **C**–**E** The expression of IL-17A, IL-17RA, and IL-17RC was measured in clinical samples using ELISA. **F**–**G** Correlation analysis of IL-17A with adiponectin and IL-1β was conducted. **H**–**I** Correlation analysis of IL-17RA with resistin and HDL-C was conducted. *IL-1β* interleukin-1 beta, *HDL-C* high-density lipoprotein cholesterol, *NC* normal control. ^*^p < 0.05, vs. FLSs; ^#^*p* < 0.05, vs. GA-FLSs; ^&^*p* < 0.05, vs. GA-PBMCs + GA-FLSs; ^△^*p* < 0.05, vs. NC; ^※^*p* < 0.05, vs. prior treatment

**Fig. 8:**
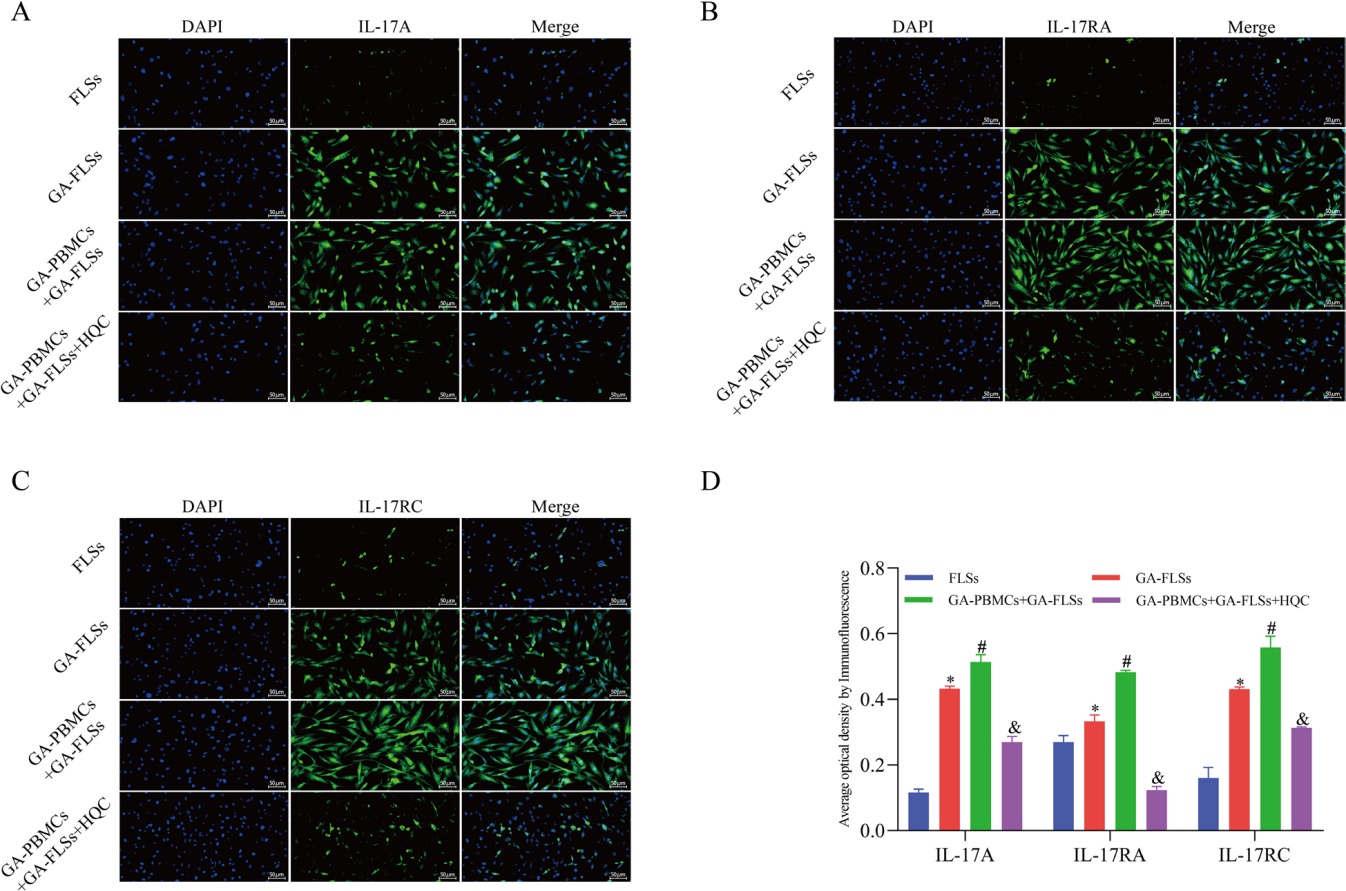
The expression of IL-17A, IL-17RA, and IL-17RC is measured through immunofluorescence (20 × ; scale bar = 50 μm). ^*^p < 0.05, vs. FLSs; ^#^*p* < 0.05, vs. GA-FLSs; ^&^*p* < 0.05, vs. GA-PBMCs + GA-FLSs

**Fig. 9:**
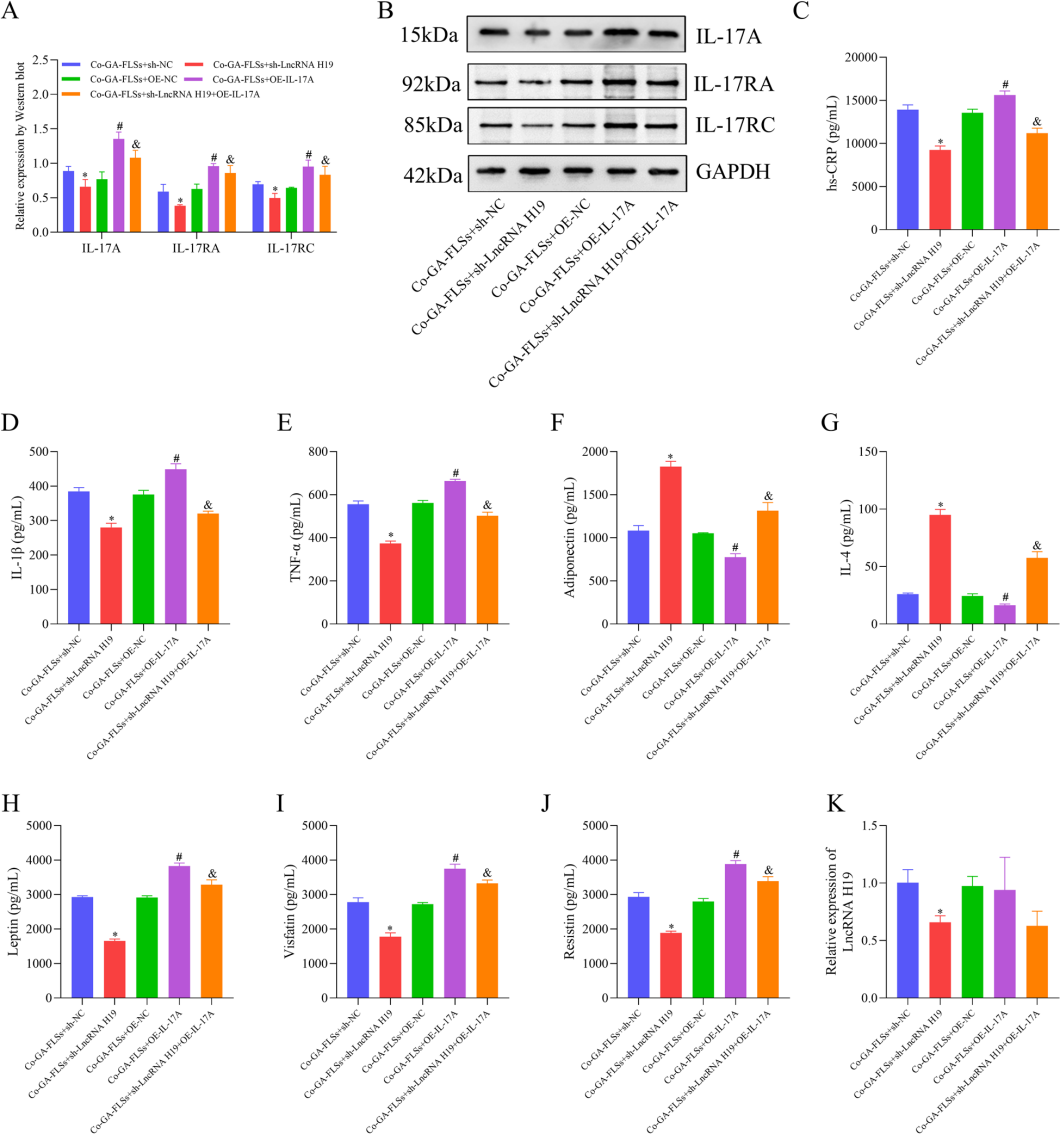
LncRNA H19 mediates GA inflammation and lipid metabolism through the IL-17 signaling pathway. **A**, **B** The expression of IL-17A, IL-17RA, and IL-17RC was measured using Western blotting. **C**–**J** The expression of hs-CRP, IL-1β, TNF-α, IL-4, adiponectin, leptin, visfatin, and resistin was measured using ELISA. **K** The expression of LncRNA H19 was measured utilizing RT-qPCR. *hs-CRP* hypersensitive C-reactive protein, *IL-1β* interleukin-1 beta, *TNF-α* tumor necrosis factor-alpha, *IL-4* interleukin-4, *LncRNA H19* long non-coding RNA H19, *Co-GA-FLSs* co-cultured GA-FLSs (GA-PBMCs + GA-FLSs). ^*^p < 0.05, vs. Co-GA-FLSs + sh-NC; ^#^*p* < 0.05, vs. Co-GA-FLSs + OE-NC; ^&^*p* < 0.05, vs. Co-GA-FLSs + sh-LncRNA H19

**Fig. 10:**
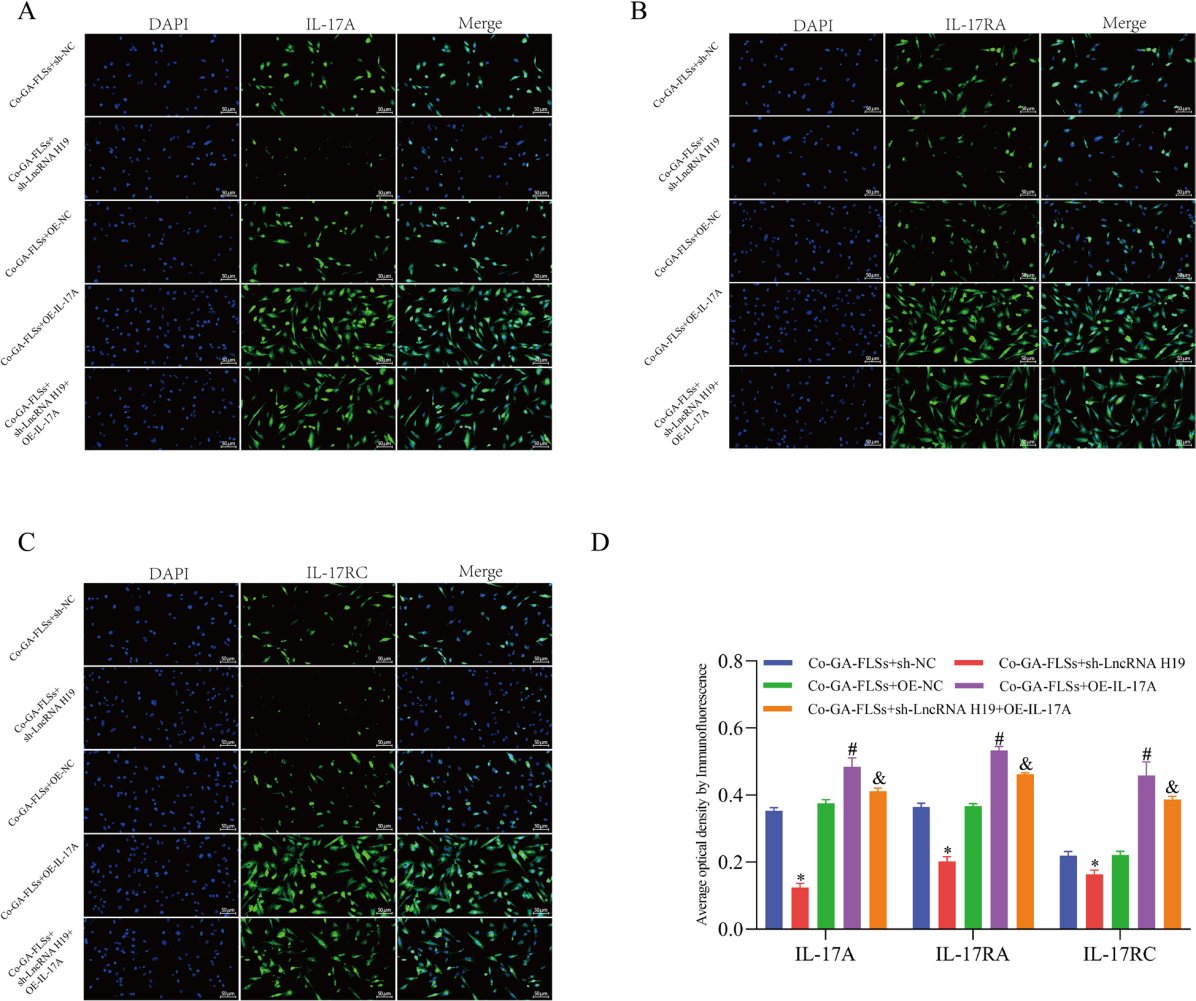
The expression of IL-17A, IL-17RA, and IL-17RC is measured through immunofluorescence (20 × ; scale bar = 50 μm). ^*^p < 0.05, vs. Co-GA-FLSs + sh-NC; ^#^*p* < 0.05, vs. Co-GA-FLSs + OE-NC; ^&^*p* < 0.05, vs. Co-GA-FLSs + sh-LncRNA H19

**Fig. 11:**
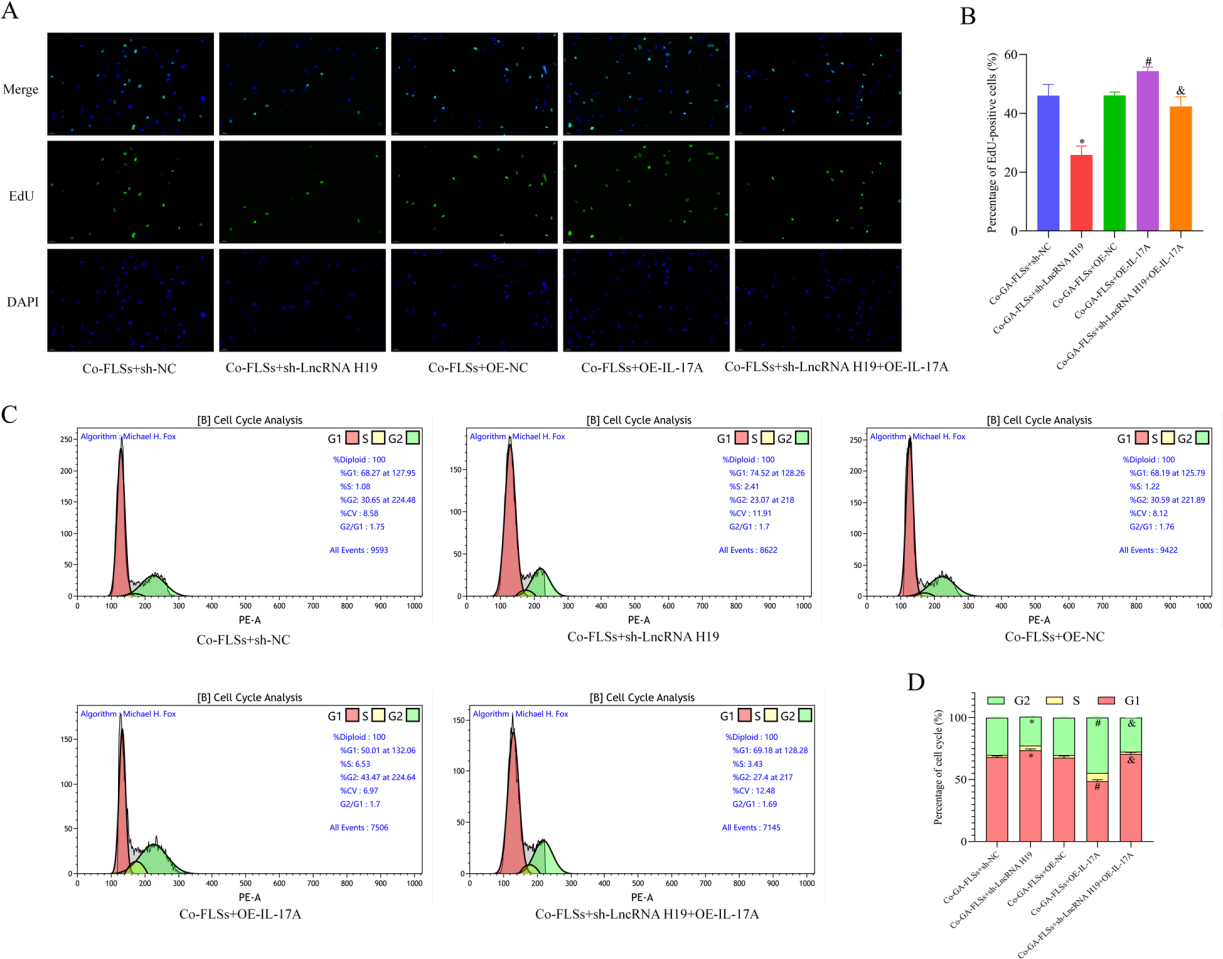
LncRNA H19 mediates the proliferation and cell cycle distribution of the co-cultured GA-FLSs through the IL-17 signaling pathway. **A**, **B** The cell proliferation was detected by EdU assay (20 × ; scale bar = 50 μm). **C**, **D** The cell cycle distribution was detected using flow cytometry. Co-GA-FLSs, co-cultured GA-FLSs (GA-PBMCs + GA-FLSs). ^*^p < 0.05, vs. Co-GA-FLSs + sh-NC; ^#^*p* < 0.05, vs. Co-GA-FLSs + OE-NC; ^&^*p* < 0.05, vs. Co-GA-FLSs + LncRNA H19

Clinical and cellular experiments were further carried out. Similar to previous findings [53], inflammatory and lipid metabolic factors were markedly disturbed in GA patients and the co-cultured GA-FLSs, which were substantially improved after HQC treatment (Table 1 and Fig. 5A–H. In addition, co-cultured GA-FLS proliferation was accelerated, with decreases in the percentage of cells at the G1 phase and obvious increases in the percentage of cells at the S/G2 phase; these results could be annulled by HQC treatment (Fig. 6A–D). Moreover, the expression of IL-17 pathway-related factors was also examined. It was found that the IL-17 pathway was over-activated in GA patients and the co-cultured GA-FLSs, which was reversed by HQC treatment (Figs. 7A–E and 8A–D). In GA patients, IL-17A and IL-17RA levels were closely related to adiponectin, IL-1β, resistin, and HDL-C levels (Fig. 7F–I). Importantly, the co-cultured GA-FLSs were transfected with OE-IL-17A plasmids. The results showed that IL-17 pathway over-activation potentiated inflammation and lipid metabolism disorders and accelerated proliferation and division in the co-cultured GA-FLSs (Figs. 9A–J, 10A–D, and 11A–D). Overall, the IL-17 pathway mediated inflammation and lipid metabolism imbalance in GA, which was an important node for HQC in ameliorating these abnormalities.

**Table 1:** Baseline characteristics of the subjects

Variables	NC (n = 20)	GA (n = 50)		Normal range	*P*_1_ Value	*P*_2_ Value
		Prior treatment	Post treatment			
Gender, male/female, n	19/1	47/3	NA	NA	0.871	NA
Age, year	59.75 ± 14.75	57.96 ± 14.87	NA	NA	0.650	NA
BMI, kg/m^2^	25.80 (23.56, 28.87)	25.01 (23.48, 27.71)	NA	NA	0.424	NA
The course of disease, year	NA	8.50 (3.00, 10.25)	NA	NA	NA	NA
hs-CRP, mg/L	NA	12.55 (3.55, 30.45)	2.20 (0.87, 4.90)	< 1	NA	< 0.001^*^
ESR, mm/h	NA	16.00 (6.75, 38.00)	6 (2.75, 10.00)	(2, 12)	NA	< 0.001^*^
TC, mmol/L	5.33 ± 0.74	5.12 ± 1.09	4.80 ± 1.18	(3, 5.7)	0.436	0.169
TG, mmol/L	1.14 ± 0.44	1.41 (0.98, 2.17)	1.10 (0.56, 1.63)	< 1.7	0.036^*^	0.006^*^
HDL-C, mmol/L	1.14 (0.94, 1.33)	1.22 (1.09, 1.34)	1.17 ± 0.30	(1.03, 1.55)	0.286	0.229
LDL-C, mmol/L	2.75 ± 0.99	3.21 ± 0.66	2.82 ± 0.85	(1.89, 4.21)	0.025^*^	< 0.011^*^
LPα, mg/L	NA	165.60 (69.28, 363.28)	NA	≤ 300	NA	NA
Apo-A1, g/L	NA	1.24 (1.10, 1.36)	NA	(1.05, 2.05)	NA	NA
Apo-B, g/L	NA	1.23 (1.02, 1.43)	NA	(0.55, 1.30)	NA	NA
BUA, μmol/L	317.20 ± 87.03	507.50 (429.25, 580.00)	417.42 ± 102.12	(154.7, 357)	< 0.001^*^	< 0.001^*^
VAS	NA	4.00 (4.00, 5.00)	1.00 (0.00, 2.00)	NA	NA	< 0.001^*^
IL-4, pg/mL	142.11 (140.87, 150.23)	31.89 (29.69, 36.97)	101.83 ± 15.13	NA	< 0.001^*^	< 0.001^*^
IL-1β, pg/mL	47.27 ± 9.89	325.25 (319.89, 354.51)	165.73 ± 13.59	NA	< 0.001^*^	< 0.001^*^
TNF-α, pg/mL	259.45 ± 14.97	898.93 ± 28.50	513.05 ± 21.19	NA	< 0.001^*^	< 0.001^*^
Adiponectin, pg/mL	2896.98 ± 138.85	744.83 (692.38, 818.53)	1727.93 (1482.36, 1834.71)	NA	< 0.001^*^	< 0.001^*^
Leptin, pg/mL	757.67 ± 74.62	3287.32 (3200.21, 3314.96)	1872.70 (1781.18, 2002.60)	NA	< 0.001^*^	< 0.001^*^
Visfatin, pg/mL	772.29 ± 89.93	2945.57 (2733.18, 3049.22)	1752.09 (1560.81, 1895.82)	NA	< 0.001^*^	< 0.001^*^
Resistin, pg/mL	629.62 ± 177.51	3714.21 (3431.48, 3863.37)	2164.23 ± 230.84	NA	< 0.001^*^	< 0.001^*^
AST, U/L	NA	20.55 (15.93, 26.93)	20.15 (16.23, 28.10)	(7, 45)	NA	0.785
ALT, U/L	NA	21.70 (14.28, 40.40)	26.00 (17.38, 41.60)	(7, 40)	NA	0.247
CREA, umol/L	NA	76.95 (67.78, 108.68)	74.40 (66.73, 86.23)	(41, 73)	NA	0.298
BUN, mmol/L	NA	5.85 (4.31, 8.26)	5.85 (4.99, 8.63)	(2.6, 7.5)	NA	0.629

*NC* normal control, *GA* gout arthritis; BMI, body mass index, *hs-CRP* hypersensitive C-reactive protein, *ESR* erythrocyte sedimentation rate, *TC* total cholesterol, *TG* triglyceride, *HDL-C* high-density lipoprotein cholesterol, *LDL-C* low-density lipoprotein cholesterin, *LPα* lipoprotein α, *Apo-A1* apolipoprotein A1, *Apo-B* apolipoprotein B, *BUA* blood uric acid, *VAS* visual analog scale, *IL-4* interleukin 4, *IL-1β* interleukin 1β, *TNF-α* tumor necrosis factor α, *AST* aspartate aminotransferase, *ALT* alanine aminotransferase, *CREA* creatinine, *BUN* blood urea nitrogen, *NA* not applicable. *P*_*1*_ Prior treatment vs. NC, *P*_*2*_ Post treatment vs. Prior treatment; ^*^P < 0.05

### The effect of the IL-17 pathway on inflammation and lipid metabolism in GA is directed by lncRNA H19

Considering the regulatory role of lncRNA H19 in GA-related inflammation and lipid metabolism disorders and the above findings, we further investigated whether lncRNA H19 drove these pathologies by modulating the IL-17 pathway. The results exhibited that lncRNA H19 knockdown blocked the activation of the IL-17 pathway, whereas IL-17 pathway activation did not affect lncRNA H19 expression (Fig. 9A, B, K and 10A–D). Through co-transfection with sh-lncRNA H19 and OE-IL-17A plasmids, it was found that activating the IL-17 pathway partially reversed the reductions in inflammation, lipid metabolism disorders, and cell proliferation and division induced by lncRNA H19 knockdown. However, this activation did not affect the decreased lncRNA H19 expression (Figs. 9A–K, 10A–D, and 11A–D). In conclusion, lncRNA H19 acted as an upstream regulator for the activation of the IL-17 pathway, thereby affecting inflammation and lipid metabolism in GA.

### HQC downregulates lncRNA H19 in GA via ALKBH5/FTO-mediated m6A modification

**Fig. 12:**
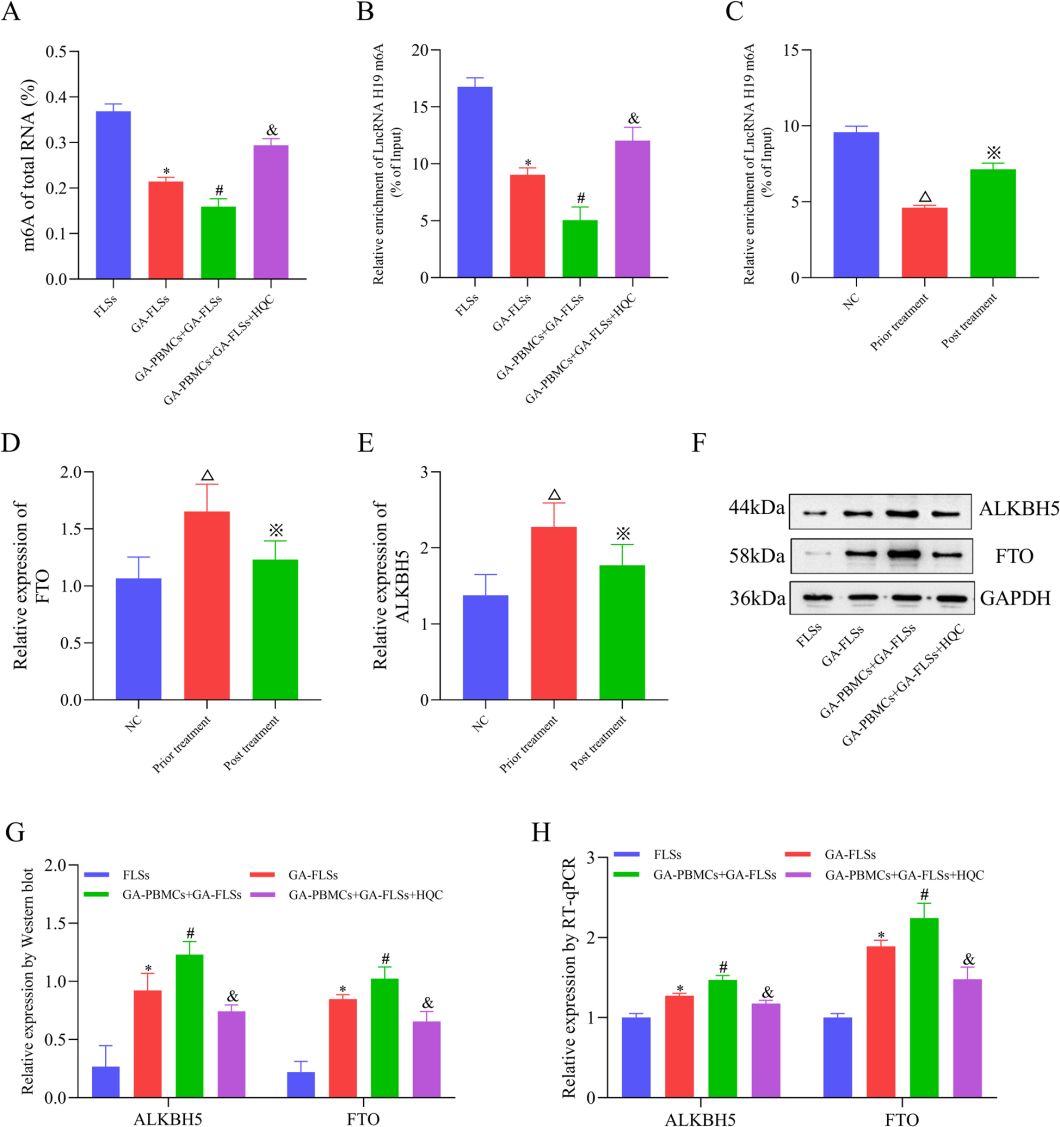
The level of m6A in GA is analyzed. **A** The level of total RNA m6A in cells was measured with colorimetric analysis. **B**, **C** The m6A level of lncRNA H19 in cells and clinical samples was measured with MeRIP-qPCR. **D**, **E** The expression of ALKBH5 and FTO in clinical samples was measured using RT-qPCR. **F**, **G** The expression of ALKBH5 and FTO in cells was measured using Western blotting. **H** The expression of ALKBH5 and FTO in cells was measured with RT-qPCR. *LncRNA H19* long non-coding RNA H19, *ALKBH5* demethylase AlkB homolog 5, *FTO* fat mass and obesity-associated protein. ^*^p < 0.05, vs. FLSs; ^#^*p* < 0.05, vs. GA-FLSs; ^&^*p* < 0.05, vs. GA-PBMCs + GA-FLSs; ^△^*p* < 0.05, vs. NC; ^※^*p* < 0.05, vs. prior treatment

**Fig. 13:**
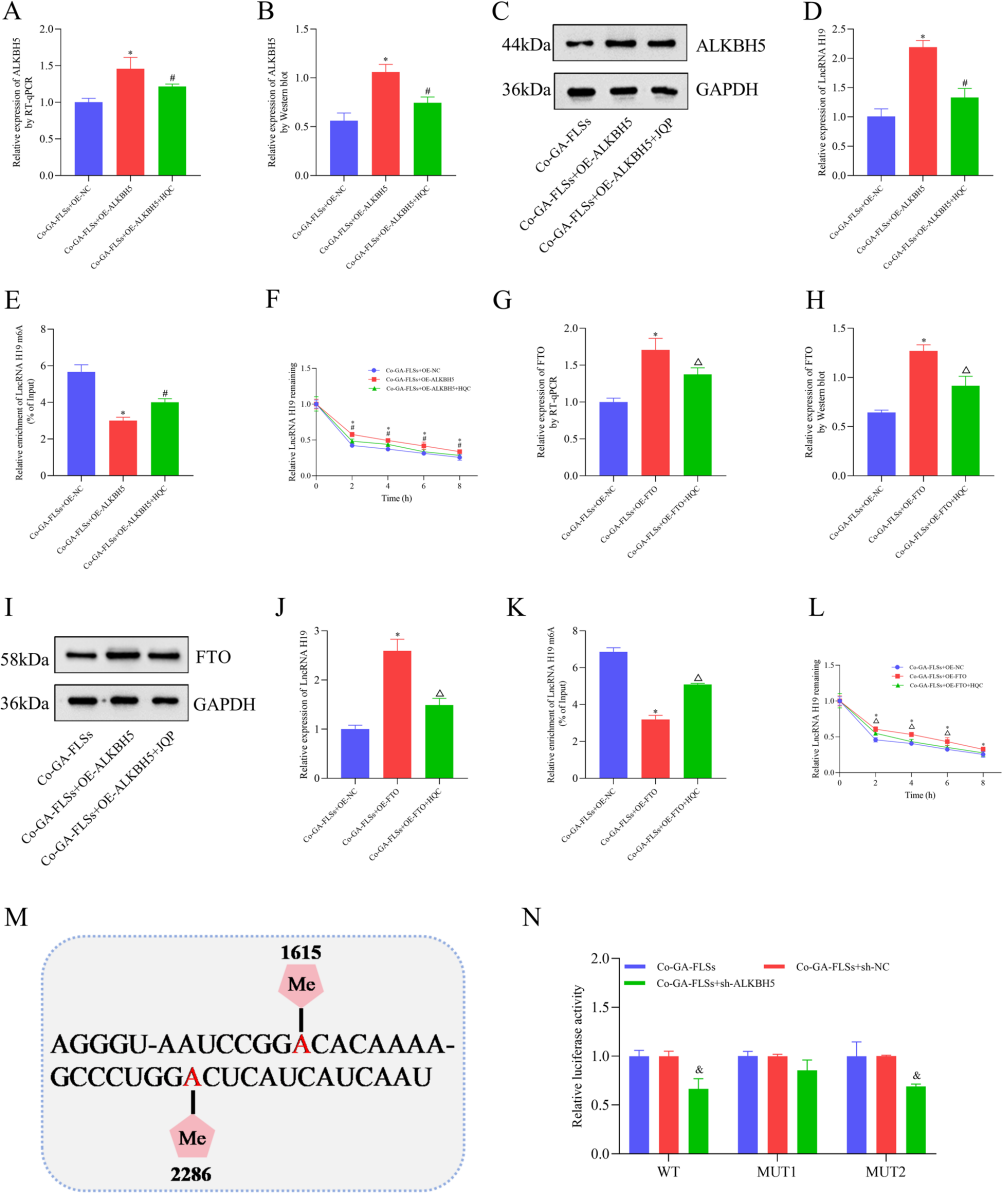
HQC regulates the expression of lncRNA H19 through ALKBH5/FTO-mediated m6A modification. **A** The expression of ALKBH5 was measured with RT-qPCR. **B**, **C** The expression of ALKBH5 was measured with Western blotting. **D** The expression of lncRNA H19 was measured via RT-qPCR. **E** The m6A level of lncRNA H19 was measured with MeRIP-qPCR. **F** The expression of lncRNA H19 was measured using RT-qPCR after 0, 2, 4, 6 and 8 h of actinomycin D treatment. **G** The expression of FTO was measured with RT-qPCR. **H**, **I** The expression of FTO was measured utilizing Western blotting. **J** The expression of lncRNA H19 was measured using RT-qPCR. **K** The m6A level of lncRNA H19 was measured with MeRIP-qPCR. **L** The expression of lncRNA H19 was measured with RT-qPCR after 0, 2, 4, 6 and 8 h of actinomycin D treatment. **M** The luciferase reporter plasmids were constructed by inserting partial wild-type lncRNA H19 sequences or mutated m6A sites (1615, 2286) lncRNA H19 sequences. **N** The luciferase reporters with wild-type or mutant plasmids were transfected into co-cultured GA-FLSs (with the interference of ALKBH5), followed by luciferase activity measurement. *LncRNA H19* long non-coding RNA H19, *ALKBH5* demethylase AlkB homolog 5, *FTO* fat mass and obesity-associated protein, *Co-GA-FLSs* co-cultured GA-FLSs (GA-PBMCs + GA-FLSs), *MUT1* mutation site 1615, *MUT2* mutation site 2286. ^*^p < 0.05, vs. Co-GA-FLSs + OE-NC; ^#^*p* < 0.05, vs. Co-GA-FLSs + OE-ALKBH5; ^△^*p* < 0.05, vs. Co-GA-FLSs + OE-FTO; ^&^*p* < 0.05, vs. Co-GA-FLSs + sh-NC

To ascertain the upstream regulatory mechanism of lncRNA H19 in GA, we measured m6A levels in GA patients and the co-cultured GA-FLSs. The results demonstrated that the total m6A level was remarkably lowered in the co-cultured GA-FLSs (Fig. 12A). Similarly, the m6A level of lncRNA H19 was declined in both GA patients and the co-cultured GA-FLSs, highlighting that the m6A modification of lncRNA H19 was notably affected in GA (Fig. 12B, C). Given that ALKBH5 and FTO are classic demethylation transferases, the present study clarified whether ALKBH5 and FTO participated in changes in the m6A modification of lncRNA H19 in GA. Markedly upregulated ALKBH5 and FTO were observed in GA patients and the co-cultured GA-FLSs (Fig. 12D − H). On this basis, ALKBH5 and FTO overexpression plasmids were constructed and transfected into the co-cultured GA-FLSs, respectively. The results revealed that ALKBH5 or FTO overexpression remarkably declined lncRNA H19 m6A levels and enhanced lncRNA H19 levels by increasing RNA stability (Fig. 13A − L). This result suggested that in GA, lncRNA H19 expression was regulated by ALKBH5/FTO-mediated m6A modification. Interestingly, it was also observed that in GA patients and cells, HQC elevated the m6A level of lncRNA H19 and reduced the expression of ALKBH5, FTO, and lncRNA H19 (Fig. 12D–H). Further rescue experiments demonstrated that HQC partially abolished the effects of ALKBH5 or FTO overexpression on lncRNA H19 m6A levels and lncRNA H19 levels (Fig. 13A–L). Taken together, HQC regulated ALKBH5/FTO-mediated m6A modification, therefore orchestrating lncRNA H19 expression in GA by affecting RNA stability. This finding illustrated the intermediate regulatory mechanism between HQC and lncRNA H19 in GA.

**Fig. 14:**
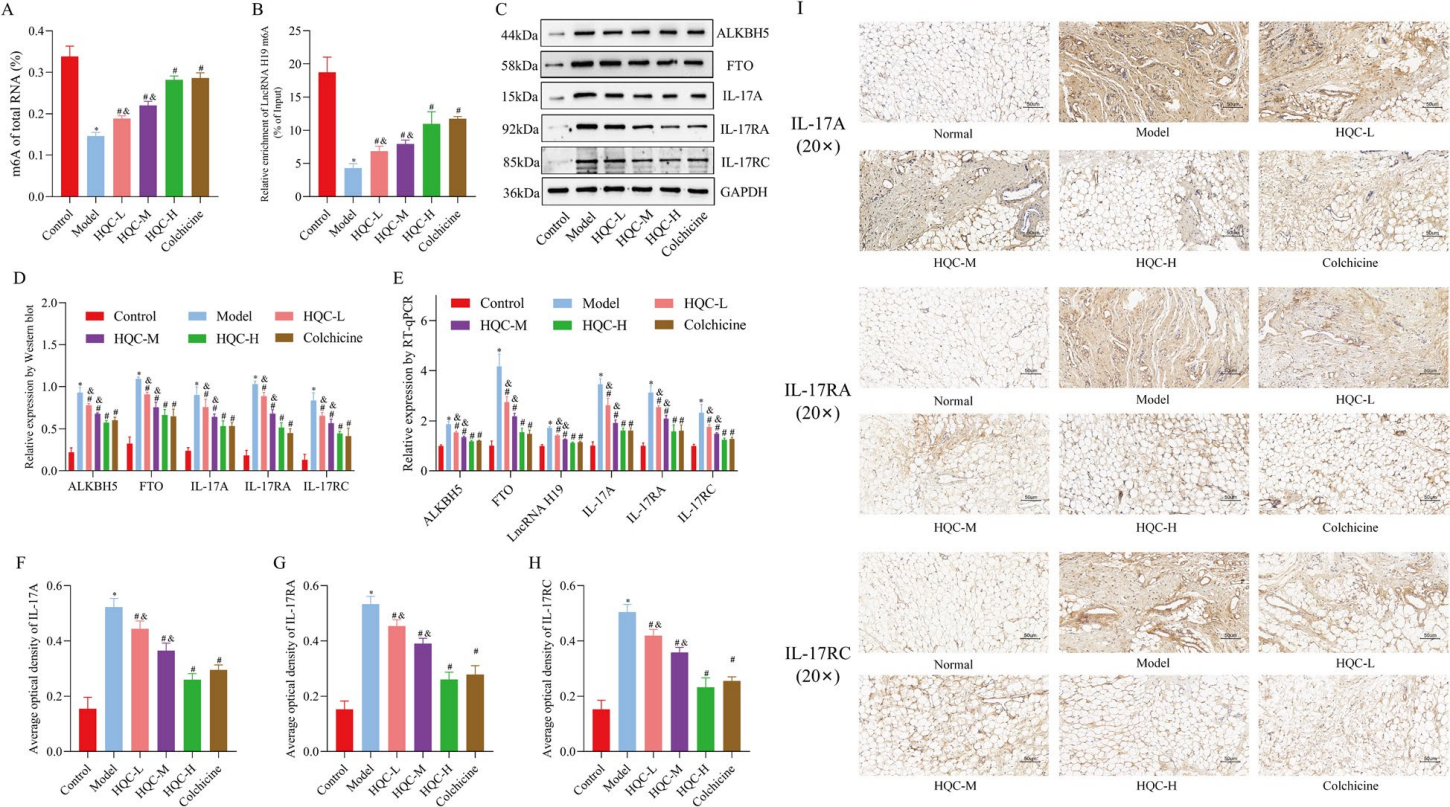
The effects of HQC on the RNA m6A, lncRNA H19, ALKBH5, FTO, and IL-17 signaling pathways in GA rats are analyzed. **A** The level of total RNA m6A was measured through colorimetric analysis. **B** The level of lncRNA H19 m6A was measured with MeRIP-qPCR. **C**, **D** The expression of ALKBH5, FTO, IL-17A, IL-17RA, and IL-17RC was measured using Western blotting. **E** The expression of ALKBH5, FTO, lncRNA H19, IL-17A, IL-17RA, and IL-17RC was measured with RT-qPCR. **F**–**I** The expression of IL-17A, IL-17RA, and IL-17RC was measured with immunohistochemistry (20 × ; scale bar = 50 μm). *LncRNA H19* long non-coding RNA H19, *ALKBH5* demethylase AlkB homolog 5, *FTO* fat mass and obesity-associated protein. ^*^p < 0.05, vs. control group; ^#^*p* < 0.05, vs. model group; ^&^*p* < 0.05, vs. colchicine group

Subsequently, relevant indicators were tested in animals to further validate clinical and cellular findings. The results demonstrated that compared with the normal group, the model group showed lower total m6A and lncRNA H19 m6A levels prominently in the synovium, but obviously higher expression of ALKBH5, FTO, IL-17A, IL-17RA, IL-17RC, and lncRNA H19 (Fig. 14A–I). Conversely, HQC treatment reversed these abnormal levels in GA rats in a dose-dependent manner (Fig. 14A–I). In summary, HQC treatment downregulated lncRNA H19 by reducing ALKBH5/FTO-mediated demethylation of lncRNA H19 m6A, thereby hindering IL-17 pathway-triggered inflammation and lipid metabolism imbalance in GA.

### The m6A modification site of lncRNA H19 is identified

Next, this study attempted to determine the exact m6A modification site of lncRNA H19. According to luciferase reporter gene assay results, the luciferase activity was decreased in the co-cultured GA-FLSs transfected with WT or MUT2 plasmids, but remained unchanged in cells transfected with MUT1 plasmids after ALKBH5 knockdown (Fig. 13M, N). Therefore, the regulation of ALKBH5 on lncRNA H19 expression mainly depended on its action on the m6A modification site of lncRNA H19 at position 1615.

### Carthamidin, the active ingredient in HQC, mediates lncRNA H19 m6A modification via ALKBH5/FTO in GA

**Fig. 15:**
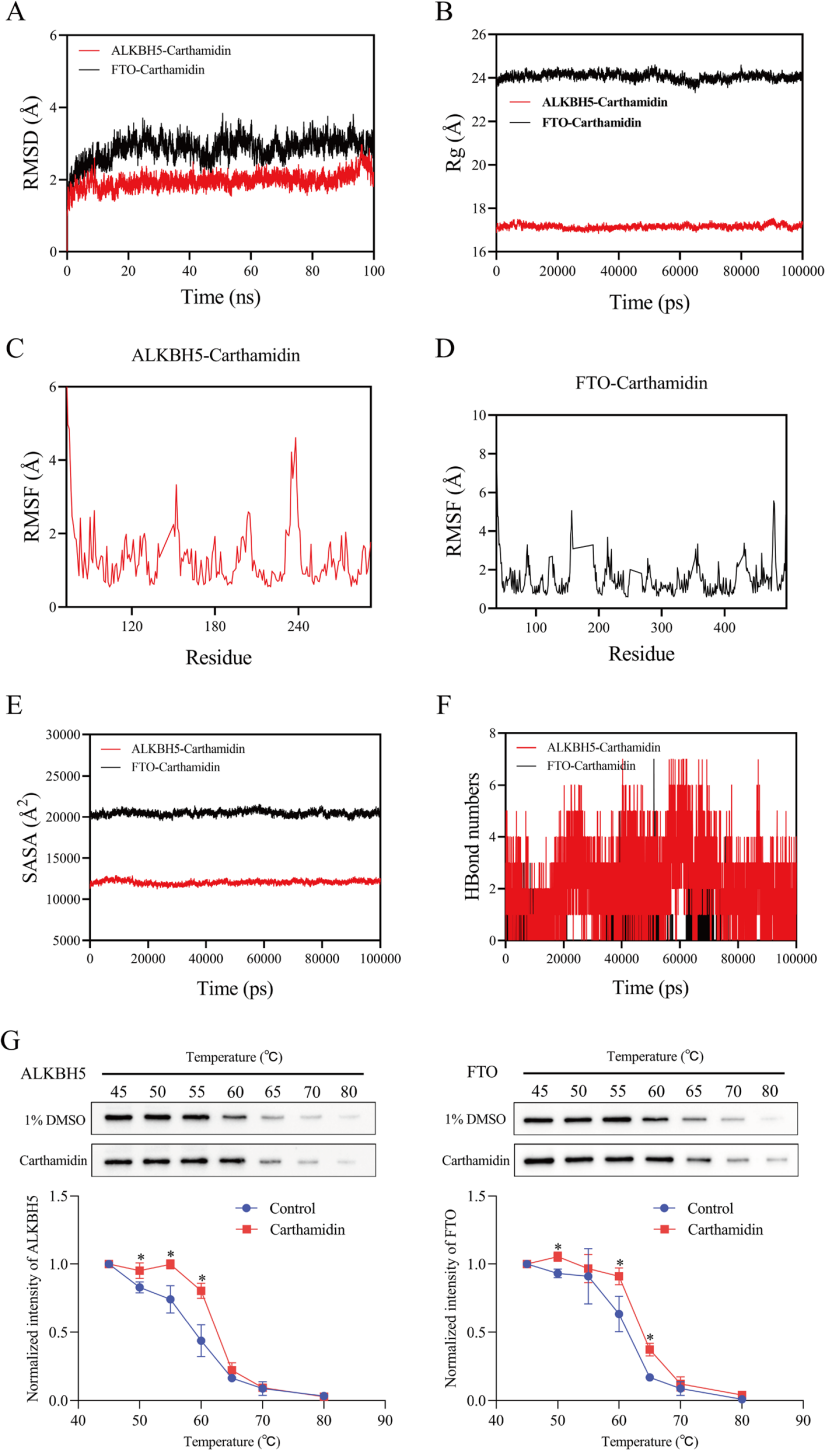
Carthamidin can be directly combined with ALKBH5 and FTO. The RMSD (**A**), Rg (**B**), RMSF (**C**, **D**), SASA (**E**), and HBonds (**F**) value of the protein–ligand complex was examined by molecular dynamics simulation. **G** The binding affinity of Carthamidin to ALKBH5 and FTO was evaluated using CETSA

Although the above work provided relatively comprehensive findings, this study further explored which active components of HQC improve inflammation and lipid metabolism in GA via ALKBH5/FTO-mediated m6A modification of lncRNA H19. The 110 active ingredients obtained by network pharmacology were intersected with 82 active ingredients of HQC identified by UPLC-Q-TOF-MS/MS [16], yielding 8 active ingredients (Table S3, Table S4, and Fig. S3). Furthermore, these 8 active ingredients were molecularly docked with ALKBH5 and FTO, respectively (Table S5). The results revealed that Carthamidin, Baicalein, Chrysin, Kaempferol, Wogonin, Quercetin, and Apigenin bound well to ALKBH5 (binding energy < -5 kcal/mol). Meanwhile, Carthamidin, Baicalein, Chrysin, Kaempferol, and Wogonin also stably bound to FTO (binding energy < -5 kcal/mol) (Fig. S4A − L). Among these ingredients, Carthamidin bound most stably to ALKBH5 and FTO (binding energy of − 7.11 and − 6.12 kcal/mol). Hence, the binding stability of Carthamidin to ALKBH5 or FTO was further assessed through molecular dynamics simulation, with RMSD, Rg, RMSF, SASA, and HBonds as parameters. In terms of the RMSD value, the ALKBH5-Carthamidin complex system reached equilibrium after 10 ns and eventually fluctuated around 2 Å, while the FTO-Carthamidin complex system reached equilibrium after 70 ns and eventually fluctuated around 2.9 Å (Fig. 15A). Regarding the Rg value, the ALKBH5-Carthamidin and FTO-Carthamidin complex systems slightly fluctuated during movement, with relatively small overall conformational changes (Fig. 15B). The RMSF values of the ALKBH5-Carthamidin and FTO-Carthamidin complexes were relatively low (mostly below 3 Å), indicating relatively poor flexibility (Fig. 15C, D). No marked changes in the SASA values of the ALKBH5-Carthamidin and FTO-Carthamidin complexes were observed, illustrating that the binding of ligands had minimal impacts on the protein structure (Fig. 15E). HBonds are vital for the binding of ligands to proteins. In most cases, approximately 5 HBonds were found in the ALKBH5-Carthamidin complex system, with about 2 HBonds in the FTO-Carthamidin complex system. This indicated good hydrogen bond interaction between this ligand and the target protein (Fig. 15F). Collectively, there was stable binding in the ALKBH5-Carthamidin and FTO-Carthamidin complex systems, and the complexes had a good hydrogen bonding effect. In addition, CETSA was conducted to verify the binding of Carthamidin to ALKBH5 or FTO. The results showed that compared with those in the control group, ALKBH5 and FTO expressions in the Carthamidin group increased prominently at different temperatures, whereas ALKBH5 and FTO expressions in the Carthamidin group were gradually lowered as temperature increased. These findings reflected that Carthamidin did not induce the degradation of ALKBH5 and FTO but improved the resistance of ALKBH5 to temperature-dependent unfolding through binding (Fig. 15G). Taken together, Carthamidin can directly target ALKBH5 and FTO, thereby conferring the effects of repressing inflammation and improving lipid metabolism in GA through downstream mechanisms.

## Discussion

GA has emerged as one of the most prevalent joint diseases worldwide, which not only induces joint pain but also leads to extra-articular lesions (heart, kidney, and blood vessel damage), significantly compromising the quality of life of patients [1]. In addition to the typical inflammatory response in GA, lipid metabolism is also gaining increasing attention. Mounting evidence shows that abnormal lipid metabolism plays an essential role in the occurrence and development of GA, such as the outbreak of joint inflammation and the occurrence of comorbidities [51]. Although lipid metabolism disturbances in GA have been identified and its possible role in GA has been gradually recognized in the past few decades, the relevant interventions remain largely unexplored, which hinders the treatment advancement of GA to a certain extent. In long-term clinical practice, TCM, which is guided by a holistic concept and syndrome differentiation, has been applied to the treatment of GA, demonstrating satisfactory outcomes [6, 59]. In particular, with the rapid development of multi-omics and multi-technology, TCM has been endowed with new and profound connotations beyond its traditional role in treating complex diseases, that is, systematic treatment through multi-components, multi-targets, and multi-pathways from a modern perspective [28]. In the context of the new era, we have conducted relevant explorations. Previous clinical and cellular studies have unraveled that high expression of lncRNA H19 drives inflammation and lipid metabolism imbalance in GA and that HQC suppresses these abnormalities in GA by decreasing lncRNA H19 expression [53]. Nevertheless, the specific mechanism remains elusive. Hence, this study investigated the role and mechanism of HQC in depressing GA-related inflammation and lipid metabolism imbalance more comprehensively.

First, a rat model of GA was established by injecting MSU into the ankle cavity of rats to evaluate the intervention effect of different doses of HQC on GA. Unsurprisingly, GA rats presented with severe inflammatory responses, accompanied by synovial and cartilage damage and joint dysfunction. Meanwhile, this study also focused on the lipid metabolism of GA rats and demonstrated that blood lipids (TC, TG, and lipoprotein A) and adipokines (adiponectin, leptin, resistin, and visfatin) were obviously disturbed in GA rats. Consistently, several previous studies [2, 19, 27] have also revealed that the lipid metabolism profile is notably changed in the serum and intestinal flora of MSU-induced GA rats, manifesting as disorders of sphingolipid, glycerophospholipid, and cholesterol metabolites. Furthermore, some researchers have pointed out that lipid metabolism disorders in GA may be caused by uric acid metabolism disorders, with a possible bidirectional causal effect between uric acid and lipid metabolism, collectively contributing to the progression of GA by provoking acute and chronic inflammation [18, 34, 46]. However, the specific mechanism remains to be explored. Additionally, the present study revealed that low, medium, and high doses of HQC inhibited inflammation, improved lipid metabolism, and mitigated synovial and cartilage damage and joint dysfunction in GA rats. In terms of safety, HQC treatment produced no obvious effects on body weight, organ index (including liver, spleen, and kidney indices), and liver and kidney function-related indicators in GA rats. These results confirmed that HQC exerted comprehensive therapeutic effects in GA rats, including anti-inflammation, lipid regulation, and improvement of joint function, with a favorable safety profile.

Network pharmacology, as an emerging discipline, is based on systems biology and biological network balance theory. It interprets the interaction between drug molecules and network targets through network node analysis and quantitatively displays key links (key molecules, key pathways, and key modules). Notably, this discipline has gradually become an effective strategy to reveal the modern connotation mechanism of TCM compounds [55]. In this study, 62 active components and 56 targets of HQC acting on inflammation and lipid metabolism in GA were identified through network pharmacology, and 10 core targets were screened out based on the PPI network. Importantly, as indicated by KEGG analysis results, among the 148 pathways enriched by the 56 targets, the IL-17 pathway ranked at the top. Additionally, 7 out of the 10 core targets were enriched in the IL-17 pathway. It has been shown that the IL-17 pathway is composed of the IL-17 family (IL-17A, IL-17B, IL-17C, IL-17D, IL-17E, and IL-17F) and its receptor family (IL-17RA, IL-17RB, IL-17RC, IL-17RD, and IL-17RE). IL-17 family members exert their functions in the form of homodimers or heterodimers by binding to IL-17 receptors [13]. IL-17A is the prototype of the IL-17 family, from which five other family members were cloned based on homology. As reported, IL-17A signals via a receptor complex composed of IL-17RA and IL-17RC to recruit nuclear factor kappa B (NF-κB) activator 1 and TNF receptor-associated factor 6. This subsequently activates NF-κB and MAPK pathways, leading to the production and release of pro-inflammatory cytokines [29]. A recent study has reported that in obese mice, the IL-17 pathway fueled lipid accumulation through NF-κB, thereby enhancing cardiac damage caused by lipid metabolism disorders [58]. IL-17A also contributes to the development of diabetic nephropathy by enhancing ectopic lipid deposition and inflammatory responses [4]. Additionally, it has been shown that monoclonal antibodies against IL-17A improve lipid metabolic profiles and have the potential to reduce cardiovascular risk in psoriasis patients [3]. The present study showed that the IL-17 pathway was substantially activated in GA patients, GA rats, and co-cultured GA-FLSs, whereas HQC treatment disrupted this pathway. Furthermore, IL-17A overexpression in the co-cultured GA-FLSs revealed that the overactivation of the IL-17 pathway promoted inflammation and lipid metabolism imbalance and accelerated proliferation and division in the co-cultured GA-FLSs. These findings underscored the important role of the IL-17 pathway in mediating inflammation and lipid metabolism in GA. Given the role of lncRNA H19 and the IL-17 pathway in GA-related inflammation and lipid metabolism disorders, this study also analyzed the relevant mechanisms. Our findings disclosed that lncRNA H19 acted as an upstream regulator to direct the activation of the IL-17 pathway, consequently affecting inflammation and lipid metabolism in GA.

As one of the most common epigenetic modifications on RNA in eukaryotes, m6A modification is dynamically modulated by three regulatory factors (methyltransferases, demethylases, and methylated reading proteins). To date, numerous studies have demonstrated the critical role of RNA m6A modification in the pathogenesis and progression of various inflammatory joint diseases, such as rheumatoid arthritis, osteoarthritis, and ankylosing spondylitis [21, 63]. However, relevant studies on GA are still lacking. Herein, the present study analyzed whether lncRNA H19 was mediated by m6A modification in GA and ascertained the related regulatory mechanism. It was found that GA patients and rats, along with the co-cultured GA-FLSs, showed significantly lowered m6A levels of lncRNA H19, suggesting changed m6A modification of lncRNA H19 in GA. As has been evidenced previously, ALKBH5 and FTO are two classic demethylases that influence RNA stability and function by specifically removing m6A modifications [15, 33]. Consistently, our results demonstrated that ALKBH5 and FTO were highly expressed in GA patients and rats, as well as in the co-cultured GA-FLSs,HQC treatment decreased ALKBH5 and FTO expressions. Additionally, the m6A level of lncRNA H19 was markedly reduced and lncRNA H19 stability was prominently elevated after overexpression of ALKBH5 or FTO in the co-cultured GA-FLSs. Further rescue experiments exhibited that HQC regulated the m6A level of lncRNA H19 through ALKBH5 and FTO and then downregulated lncRNA H19 by compromising RNA stability.

Carthamidin is present in a bewildering array of natural plants, which was predominantly utilized in ancient times for producing rouge as one of the essential dyes [11, 60]. Recently, the pharmacological effect of Carthamidin has been progressively discovered [57]. For instance, a prior study has reported that Carthamidin strongly impedes MCF7 cell proliferation and holds significant therapeutic potential in breast cancer [31]. In this study, the active ingredients of HQC were identified using network pharmacology and then intersected with the active ingredients identified by UPLC-Q-TOF-MS/MS. According to molecular docking results, among the intersected active ingredients, Carthamidin bound to ALKBH5 and FTO in the most stable way. Finally, CETSA results confirmed that Carthamidin directly targeted ALKBH5 and FTO, therefore contributing to repressing inflammation and improving lipid metabolism in GA treatment through downstream mechanisms.

Collectively, our study unravels a new mechanism of HQC in repressing inflammation and improving lipid metabolism in GA from clinical, cellular, and animal aspects. The findings of this article may provide further support for the clinical application of HQC in GA treatment. However, there are some limitations in this study. First, network pharmacological analysis identified many pathways involved in the effect of HQC on inflammation and lipid metabolism in GA. Nevertheless, this study only focused on the IL-17 pathway, which may overlook the possibility of other pathways. Second, lncRNA H19 m6A modification in GA may be mediated not only by ALKBH5 and FTO. Accordingly, further investigations are warranted to identify the specific methyltransferases and methylated reading protein regulating lncRNA H19 m6A modification in GA. Third, the overall efficacy of HQC may result from the combined action of multiple components, and Carthamidin is just one of them. Additional studies are necessary to explore other active ingredients of HQC that improve inflammation and lipid metabolism in GA. Fourth, further *in-vitro* and *in-vivo* experiments are needed to analyze the inflammation- and lipid-regulating effects of Carthamidin in GA treatment. Fifth, this study only included 20 normal individuals and 50 GA patients, which may restrict the statistical power of this study, particularly for evaluating the effects of drug interventions and the correlation analysis. Sixth, there remains a lack of gene knockout studies on ALKBH5, FTO, lncRNA H19, lncRNA H19 m6A, and the IL-17 pathway in animal models. Future studies should address these research limitations to provide additional evidence elucidating GA pathogenesis and to support the application of TCM in the prevention and treatment of GA.

## Conclusion

**Fig. 16:**
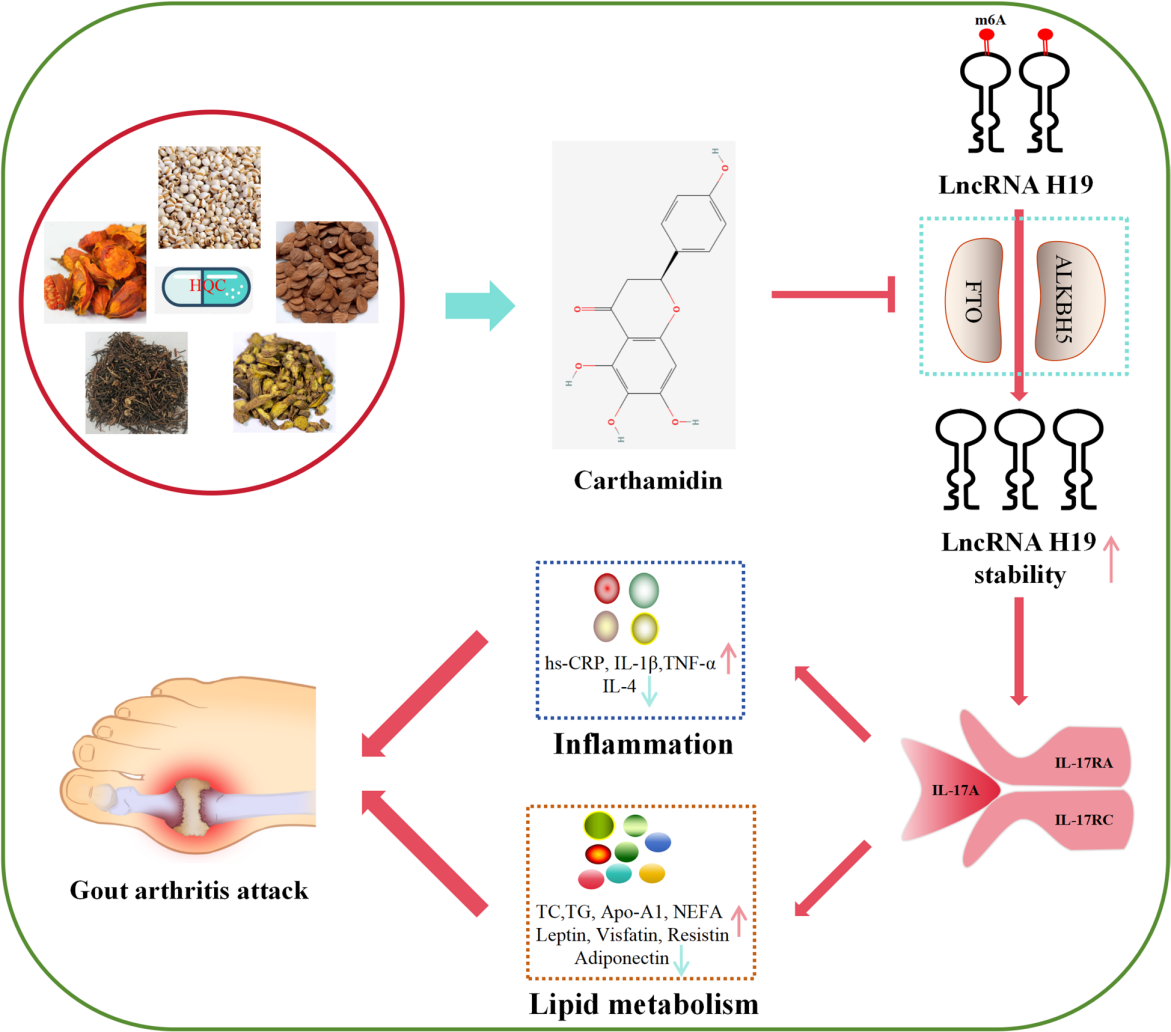
HQC downregulated lncRNA H19 levels by reducing ALKBH5/FTO-mediated demethylation of its m6A modifications, thereby decreasing RNA stability. This subsequently improves GA inflammation and lipid metabolism imbalance driven by the IL-17 signaling pathway

Conclusively, overexpression of ALKBH5 and FTO decreases the m6A modification level of lncRNA H19, leading to its upregulation and subsequent activation of the IL-17 pathway, which potentiates inflammation and lipid metabolism imbalance in GA. Conversely, HQC reverses this process by decreasing ALKBH5/FTO-mediated m6A demethylation of lncRNA H19 and then compromising RNA stability, thereby suppressing IL-17 pathway-driven pathology. This effect of HQC is partly ascribed to Carthamidin in HQC (Fig. 16). Overall, our findings highlight that HQC is a promising drug for the treatment of GA.

## Supplementary Information

Additional file 1.

Additional file 2.

Additional file 3.

Additional file 4.

Additional file 5.

Additional file 6.

Additional file 7.

Additional file 8.

Additional file 9.

Additional file 10.

## Data Availability

No datasets were generated or analysed during the current study.

## Abbreviations

ALKBH5: AlkB homolog 5
AI: Arthritis index
ALT: Alanine aminotransferase
AST: Aspartate aminotransferase
BUA: Blood uric acid
BUN: Blood urea nitrogen
CETSA: Cellular thermal shift assay
CREA: Creatinine
EdU: 5-Ethynyl-2’-deoxyuridine
ELISA: Enzyme-linked immunosorbent assay
ESR: Erythrocyte sedimentation rate
FCM: Flow cytometry
FDI: Foot dysfunction index
FLSs: Fibroblast-like synoviocytes
FTO: Fat mass and obesity-associated protein
GA: Gouty arthritis
HE: Hematoxylin–eosin
HDL-C: High-density lipoprotein cholesterol
hs-CRP: Hypersensitive C-reactive protein
IL: Interleukin
HQC: Huangqin Qingrechubi Capsule
LDL-C: Low-density lipoprotein cholesterol
LncRNA: Long non-coding RNA
m6A: N6-methyladenosine
MSU: Monosodium urate
PBMCs: Peripheral blood mononuclear cells
RT-qPCR: Real-time quantitative polymerase chain reaction
TC: Total cholesterol
TEM: Transmission electron microscopy
TG: Triglyceride
TNF: Tumor necrosis factor
UPLC-Q-TOF–MS/MS: Ultra-performance liquid chromatography-quadrupole time-of-flight tandem mass spectrometry
VAS: Visual analog scale
