# Supplementary material for: The IL-17 pathway mediated by m6A-modified lncRNA H19: a new mechanism for Jianpi Qingre Tongluo Prescription in repressing inflammation and improving lipid metabolism in gout arthritis
Source: Chin Med. 2026 Mar 18;21:95. doi: 10.1186/s13020-026-01379-z (PMC12997696; doi:10.1186/s13020-026-01379-z)
Supplement: Supplementary file 1 — Additional file 1. [file 13020_2026_1379_MOESM1_ESM.docx]

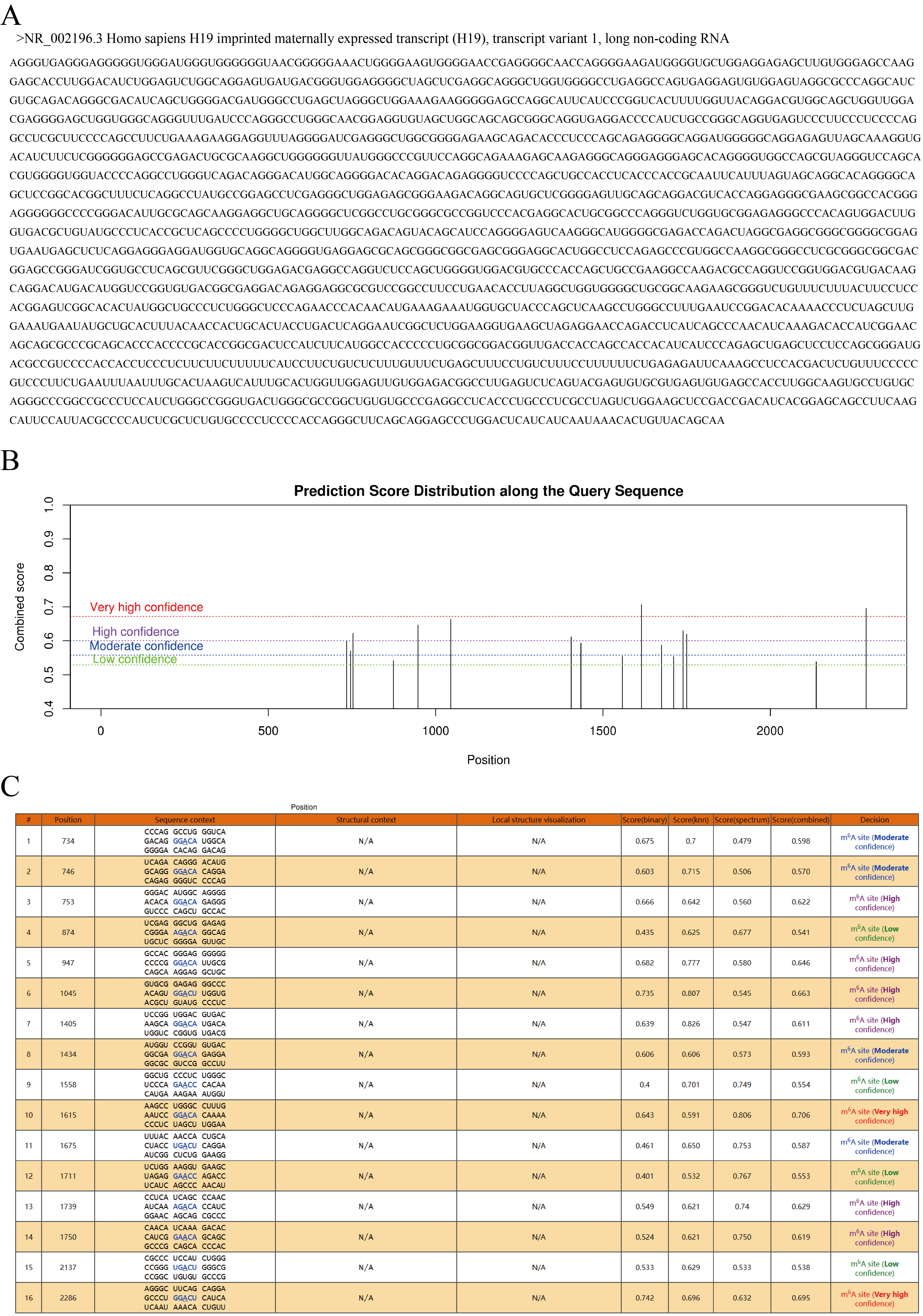


**Supplementary Figure 1** SRAMP software (http://www.cuilab.cn/sramp/) predicted the possible m6A methylation sites of LncRNA H19. (A) The transcript sequence of LncRNA H19. (B) Prediction score distribution map of m6A modification sites of LncRNA H19. (C) Prediction information table of m6A modification sites of LncRNA H19. LncRNA H19, long non-coding RNA H19.


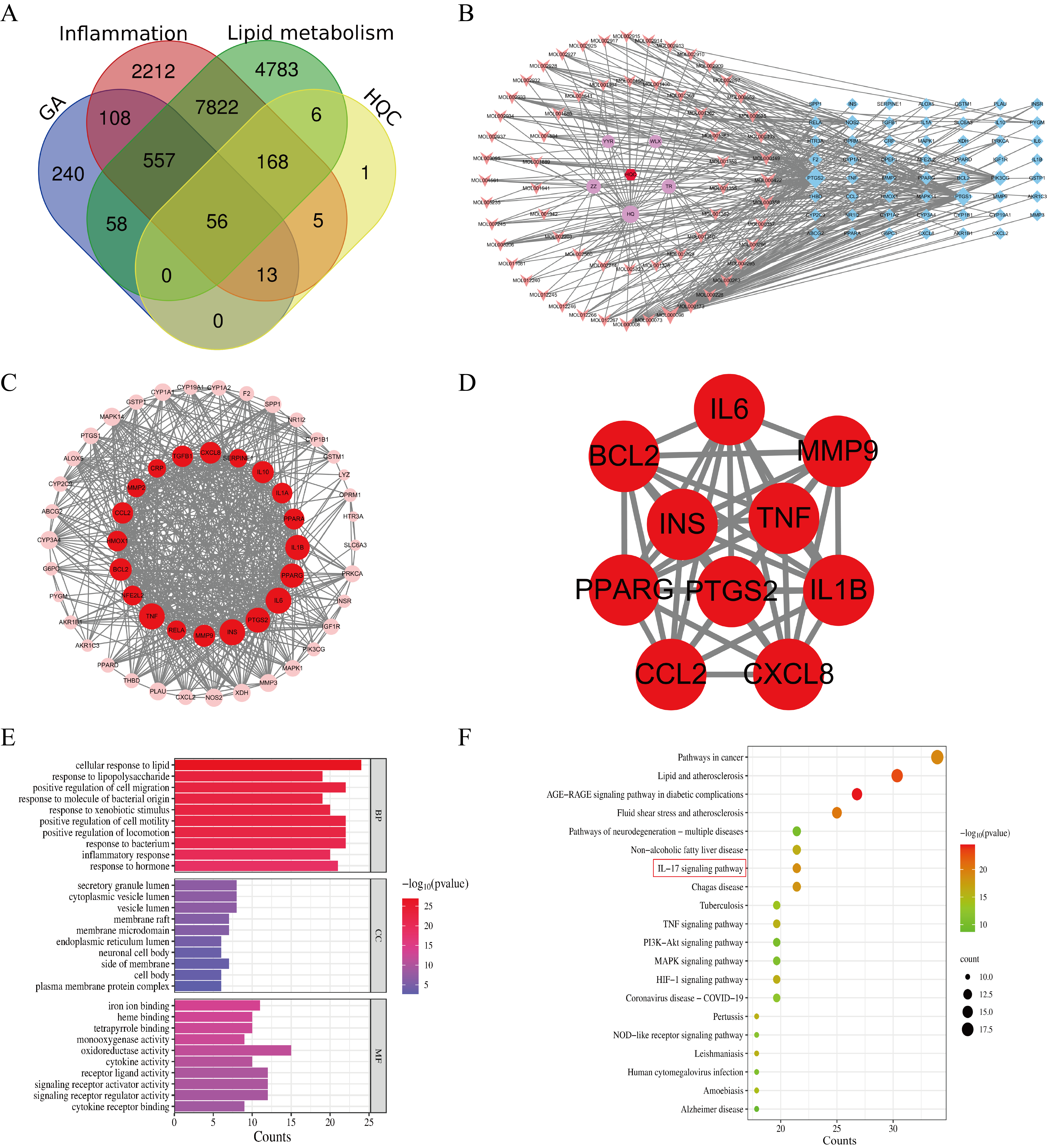


**Supplementary Figure 2** Network pharmacological analysis of the effects of HQC on GA-induced inflammation and lipid Metabolism. (A) Venn diagram of target genes of the drug and disease. Among them, the number of genes related to HQC, GA, inflammation and lipid metabolism were 249, 1032, 10941 and 13450, respectively. After taking the intersection, a total of 56 common genes were finally obtained. (B) Drug-active ingredient-target network. The regular hexagon in the middle of the left side is HQC, which is the Huangqin Qingrechubi Capsule. The circle on the left represents the drug, where HQ is Huangqin, ZZ is Zhizi, YYR is Yiyiren, TR is a Taoren, and WLX is Weilingxian. The triangle on the left represents the active ingredient. The right diamond is the target gene, and the larger the area, the higher the importance. (C) Protein-protein interaction (PPI) network. It contains 55 nodes and 602 edges (one of the target genes was a free point), and the larger the area, the higher the importance. (D) The top 10 core targets according to degree value. It contains 10 nodes and 45 edges. (E) Gene ontology (GO) enrichment analysis. Biological process (BP), cellular component (CC) and molecular functions (MF) are enriched here. The ordinate is the project description, and the abscissa is the number of targets enriched. The color represents different p values, and the redder the color, the more significant it is. (F) Kyoto encyclopedia of genes and genomes (KEGG) enrichment analysis. The ordinate is the project description, and the abscissa is the number of targets enriched. The color represents different p values, and the redder the color, the more significant it is.


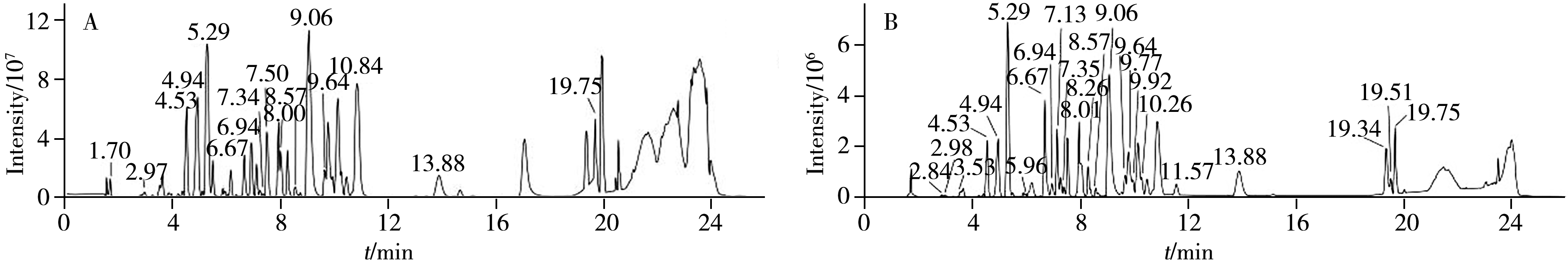


**Supplementary Figure 3** Base peak chromatograms of HQC in positive (A) and negative (B) ion modes. The ordinate is the intensity, and the abscissa is the time. The specific mass spectrometry data and identification results are presented in Table S4.


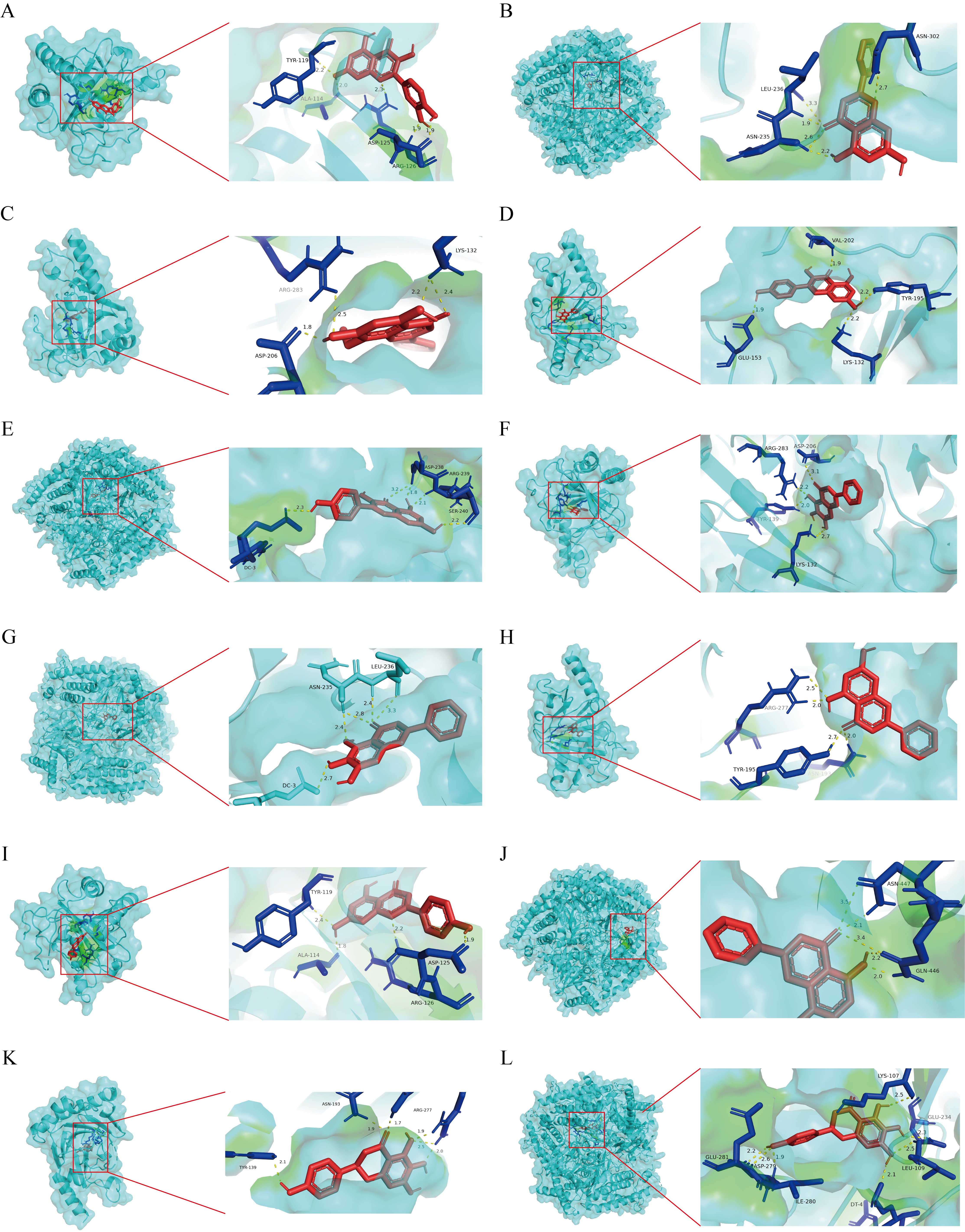


**Supplementary Figure 4** Visualization of the docking of active ingredients with ALKBH5 and FTO. (A) The diagram of docking between Quercetin (MOL000098) and ALKBH5 (4NRM). (B) The diagram of docking between Chrysin (MOL002560) and FTO (5ZMD). (C) The diagram of docking between Wogonin (MOL000173) and ALKBH5 (4NRM). (D) The diagram of docking between Kaempferol (MOL000422) and ALKBH5 (4NRM). (E) The diagram of docking between Kaempferol (MOL000422) and FTO (5ZMD). (F) The diagram of docking between Baicalein (MOL002714) and ALKBH5 (4NRM). (G) The diagram of docking between Baicalein (MOL002714) and FTO (5ZMD). (H) The diagram of docking between Chrysin (MOL002560) and ALKBH5 (4NRM). (I) The diagram of docking between Apigenin (MOL000008) and ALKBH5 (4NRM). (J) The diagram of docking between Wogonin (MOL000173) and FTO (5ZMD). (K) The diagram of docking between Carthamidin (MOL002910) and ALKBH5 (4NRM). (L) The diagram of docking between Carthamidin (MOL002910) and FTO (5ZMD). ALKBH5, AlkB homolog 5; FTO, fat mass and obesity-associated protein.
