# Supplementary figures and images for: The IL-17 pathway mediated by m6A-modified lncRNA H19: a new mechanism for Jianpi Qingre Tongluo Prescription in repressing inflammation and improving lipid metabolism in gout arthritis
Source: Chin Med. 2026 Mar 18;21:95. doi: 10.1186/s13020-026-01379-z (PMC12997696; doi:10.1186/s13020-026-01379-z)

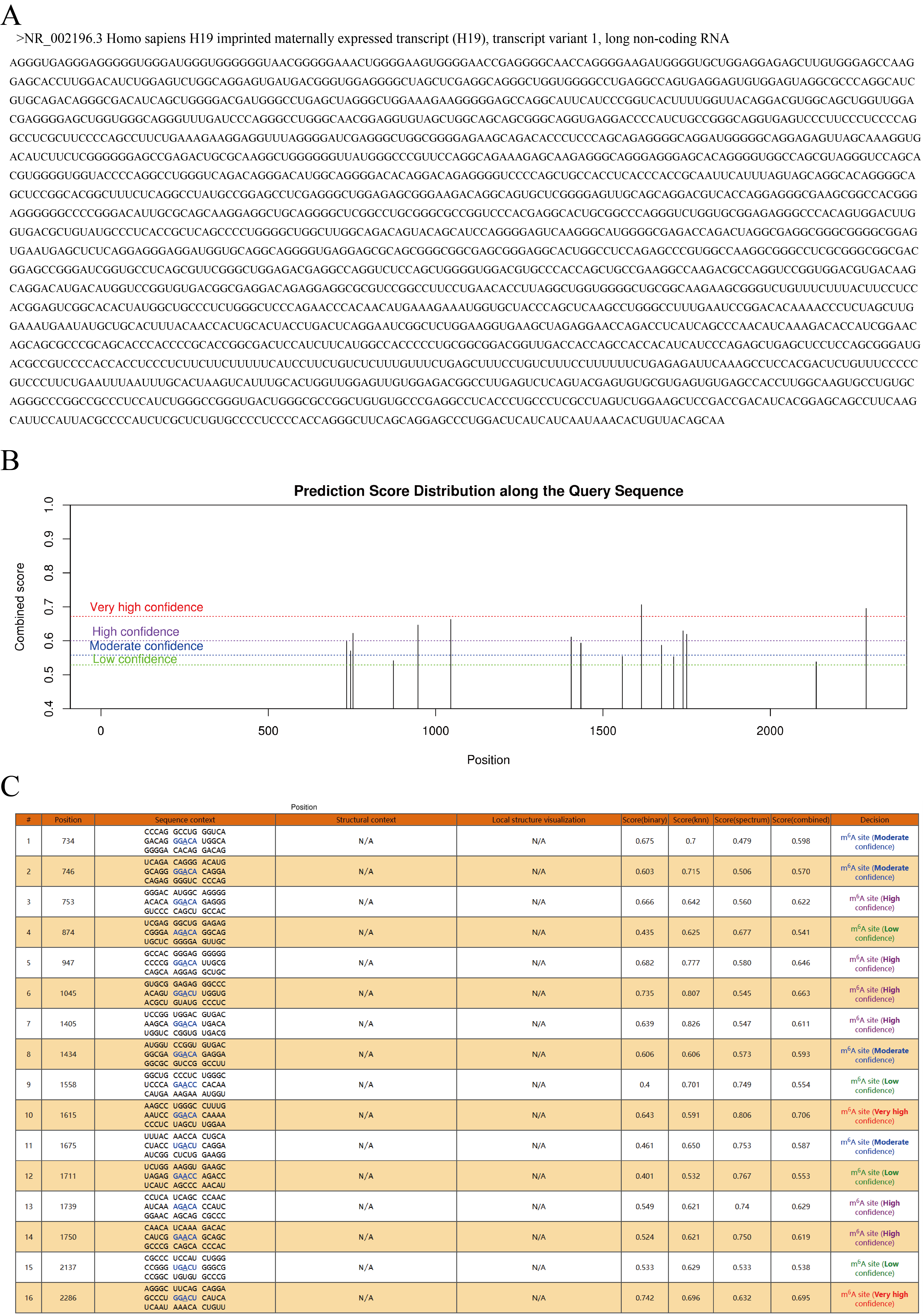

Supplement: Supplementary file 2 — Additional file 2. [file 13020_2026_1379_MOESM2_ESM.png]

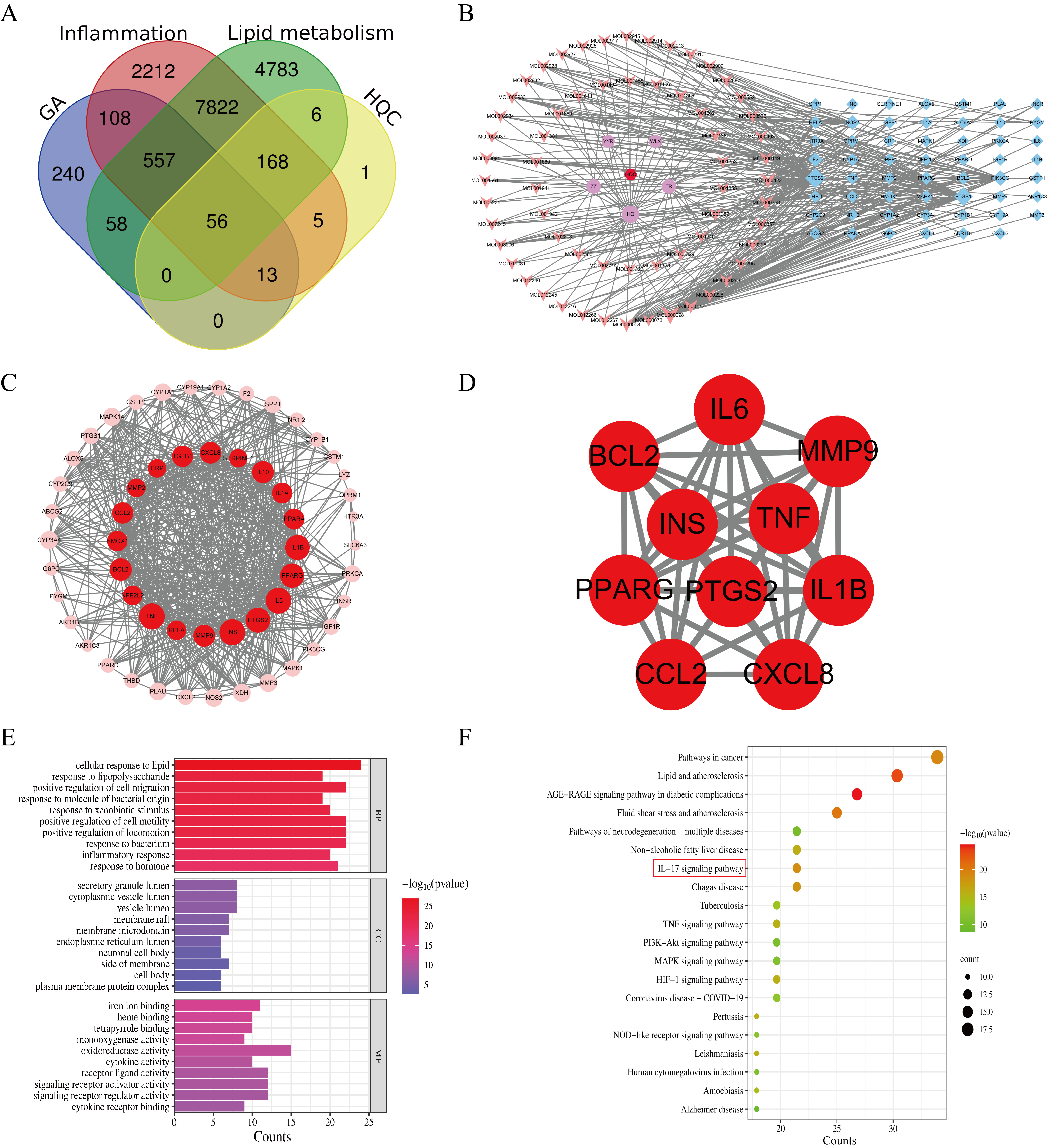

Supplement: Supplementary file 3 — Additional file 3. [file 13020_2026_1379_MOESM3_ESM.png]

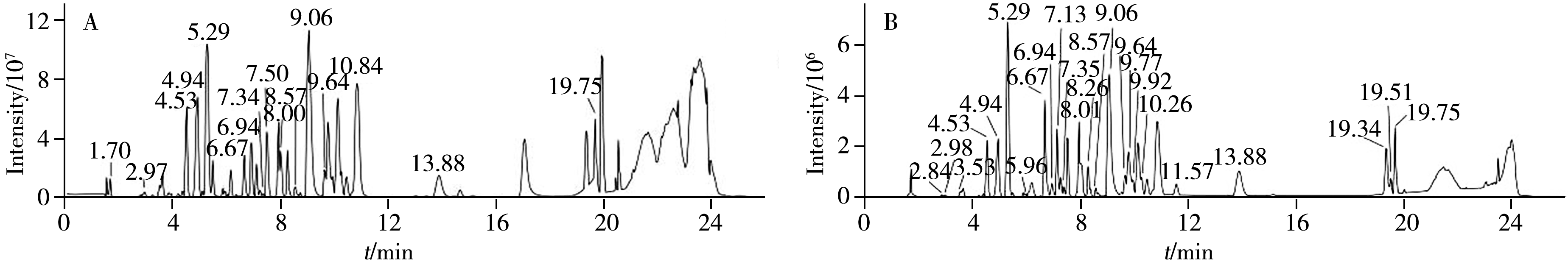

Supplement: Supplementary file 4 — Additional file 4. [file 13020_2026_1379_MOESM4_ESM.tif]
