# Supplementary material for: The IL-17 pathway mediated by m6A-modified lncRNA H19: a new mechanism for Jianpi Qingre Tongluo Prescription in repressing inflammation and improving lipid metabolism in gout arthritis
Source: Chin Med. 2026 Mar 18;21:95. doi: 10.1186/s13020-026-01379-z (PMC12997696; doi:10.1186/s13020-026-01379-z)
Supplement: Supplementary file 6 — Additional file 6. [file 13020_2026_1379_MOESM6_ESM.docx]

**Supplementary table 1** Primer sequences used in the study

| Target | Amplicon size（bp） | Primer sequence (5′-3′) |
| --- | --- | --- |
| Hsa-LncRNA H19 | 82 | F: GCGGGTCTGTTTCTTTACTTCC  R: TCTTTCATGTTGTGGGTTCTGG |
| Hsa-ALKBH5 | 81 | F: GGAATGTCTTCTTGTCAGCC  R: AAGTGGTGGTATCCTGGTTG |
| Hsa-FTO | 141 | F: CGCGAAGCTAAGAAACTGAG  R: AGATACACTGCTGGCTTCTC |
| Hsa-β-actin | 96 | F: CCCTGGAGAAGAGCTACGAG  R: GGAAGGAAGGCTGGAAGAGT |
| Hsa-LncRNA H19 shRNA 1# | NA | F: GCGGGUCUGUUUCUUUACUTT  R: AGUAAAGAAACAGACCCGCTT |
| Hsa-LncRNA H19 shRNA 2# | NA | F: CCCGUCCCUUCUGAAUUUATT  R: UAAAUUCAGAAGGGACGGGTT |
| Hsa-LncRNA H19 shRNA 3# | NA | F: CUGGACUCAUCAUCAAUAATT  R: UUAUUGAUGAUGAGUCCAGTT |
| Hsa-ALKBH5 shRNA 1# | NA | F: GCUGCAAGUUCCAGUUCAAGC  R: UUGAACUGGAACUUGCAGCCG |
| Hsa-ALKBH5 shRNA 2# | NA | F: GCUUCAGCUCUGAGAACUACU  R: UAGUUCUCAGAGCUGAAGCUA |
| Hsa-ALKBH5 shRNA 3# | NA | F: GGACCUAGGUUCUCAUAUUCU  R: AAUAUGAGAACCUAGGUCCUG |
| Rat-LncRNA H19 | 127 | F: ACCTCGGCATCTGGAGT  R: GTTTTCCAGCCTCTGCAATG |
| Rat-ALKBH5 | 108 | F: GGGTATGCTGCTGATGAGAT  R: TTCCAATCGTGGTGCATCTA |
| Rat-FTO | 123 | F: TATCTTACAACGCTGCCAGT  R: ACCTCGTTGTGGATCTCTTC |
| Rat-IL-17A | 161 | F: ATTCCATCCATGTGCCTGAT  R: GAGCTAAGGGAGTTGAGGAC |
| Rat-IL-17RA | 153 | F: TCAGTCCTCGGCTAGGAAA  R: AACAGCAGAAGCAGCCAT |
| Rat-IL-17RC | 149 | F: TTTCAGGCCTACCCCATTG  R: TTGGTGTAGGACCAGATTCG |
| Rat-β-actin | 150 | F: CCCATCTATGAGGGTTACGC  R: TTTAATGTCACGCACGATTTC |

Hsa, homo sapiens; LncRNA H19, long non-coding RNA H19; ALKBH5, AlkB homolog 5; FTO, fat mass and obesity-associated protein; shRNA, short hairpin RNA; IL-17A, interleukin 17A; IL-17RA, interleukin 17 receptor A; IL-17RC, interleukin 17 receptor C; F, forward; R, reverse; NA, not applicable.
