# Supplementary material for: The IL-17 pathway mediated by m6A-modified lncRNA H19: a new mechanism for Jianpi Qingre Tongluo Prescription in repressing inflammation and improving lipid metabolism in gout arthritis
Source: Chin Med. 2026 Mar 18;21:95. doi: 10.1186/s13020-026-01379-z (PMC12997696; doi:10.1186/s13020-026-01379-z)
Supplement: Supplementary file 7 — Additional file 7. [file 13020_2026_1379_MOESM7_ESM.docx]

**Supplementary table 2** Disease database information

| Database | Website address |
| --- | --- |
| Online Mendelian Inheritance in Man (OMIM) | https://www.omim.org/ |
| Gene Database (GeneCards) | https://www.genecards.org/ |
| DrugBank Database | https://www.drugbank.ca/ |
| Therapeutic Target Database (TTD) | http://db.idrblab.net/ttd/ |
| DisGeNeT Database | https://www.disgenet.org/ |
| PharmGKB | https://www.pharmgkb.org/ |
