# Supplementary material for: The IL-17 pathway mediated by m6A-modified lncRNA H19: a new mechanism for Jianpi Qingre Tongluo Prescription in repressing inflammation and improving lipid metabolism in gout arthritis
Source: Chin Med. 2026 Mar 18;21:95. doi: 10.1186/s13020-026-01379-z (PMC12997696; doi:10.1186/s13020-026-01379-z)
Supplement: Supplementary file 8 — Additional file 8. [file 13020_2026_1379_MOESM8_ESM.docx]

**Supplementary table 3** Active ingredient information of HQC from network pharmacology

| TCM | No. | MOL ID | Name of molecular | OB (%) | DL |
| --- | --- | --- | --- | --- | --- |
| HQ | 1 | MOL002934 | NEOBAICALEIN | 104.34 | 0.44 |
|  | 2 | MOL002932 | Panicolin | 76.26 | 0.29 |
|  | 3 | MOL012246 | 5,7,4'-trihydroxy-8-methoxyflavanone | 74.24 | 0.26 |
|  | 4 | MOL002927 | Skullcapflavone II | 69.51 | 0.44 |
|  | 5 | MOL002911 | 2,6,2',4'-tetrahydroxy-6'-methoxychaleone | 69.04 | 0.22 |
|  | 6 | MOL002937 | DIHYDROOROXYLIN | 66.06 | 0.23 |
|  | 7 | MOL000228 | (2R)-7-hydroxy-5-methoxy-2-phenylchroman-4-one | 55.23 | 0.20 |
|  | 8 | MOL002915 | Salvigenin | 49.07 | 0.33 |
|  | 9 | MOL000073 | ent-Epicatechin | 48.96 | 0.24 |
|  | 10 | MOL011081 | Linolenic acid methyl ester | 46.15 | 0.17 |
|  | 11 | MOL002917 | 5,2',6'-Trihydroxy-7,8-dimethoxyflavone | 45.05 | 0.33 |
|  | 12 | MOL000449 | Stigmasterol | 43.83 | 0.76 |
|  | 13 | MOL008206 | Moslosooflavone | 44.09 | 0.25 |
|  | 14 | MOL002879 | Diop | 43.59 | 0.39 |
|  | 15 | MOL001490 | bis[(2S)-2-ethylhexyl] benzene-1,2-dicarboxylate | 43.59 | 0.35 |
|  | 16 | MOL002897 | epiberberine | 43.09 | 0.78 |
|  | 17 | MOL001889 | Methyl linolelaidate | 41.93 | 0.17 |
|  | 18 | MOL002928 | oroxylin a | 41.37 | 0.23 |
|  | 19 | MOL002914 | Eriodyctiol (flavanone) | 41.35 | 0.24 |
|  | 20 | MOL002910 | Carthamidin | 41.15 | 0.24 |
|  | 21 | MOL002913 | Dihydrobaicalin_qt | 40.04 | 0.21 |
|  | 22 | MOL000525 | Norwogonin | 39.40 | 0.21 |
|  | 23 | MOL010415 | 11,13-Eicosadienoic acid, methyl ester | 39.28 | 0.23 |
|  | 24 | MOL002926 | dihydrooroxylin A | 38.72 | 0.23 |
|  | 25 | MOL012266 | rivularin | 37.94 | 0.37 |
|  | 26 | MOL000359 | sitosterol | 36.91 | 0.75 |
|  | 27 | MOL000358 | beta-sitosterol | 36.91 | 0.75 |
|  | 28 | MOL002908 | 5,8,2'-Trihydroxy-7-methoxyflavone | 37.01 | 0.27 |
|  | 29 | MOL002925 | 5,7,2',6'-Tetrahydroxyflavone | 37.01 | 0.24 |
|  | 30 | MOL012245 | 5,7,4'-trihydroxy-6-methoxyflavanone | 36.63 | 0.27 |
|  | 31 | MOL002933 | 5,7,4'-Trihydroxy-8-methoxyflavone | 36.56 | 0.27 |
|  | 32 | MOL001689 | acacetin | 34.97 | 0.24 |
|  | 33 | MOL002909 | 5,7,2,5-tetrahydroxy-8,6-dimethoxyflavone | 33.82 | 0.45 |
|  | 34 | MOL001506 | Supraene | 33.55 | 0.42 |
|  | 35 | MOL002714 | baicalein | 33.52 | 0.21 |
|  | 36 | MOL000552 | 5,2'-Dihydroxy-6,7,8-trimethoxyflavone | 31.71 | 0.35 |
|  | 37 | MOL001458 | coptisine | 30.67 | 0.86 |
|  | 38 | MOL000173 | wogonin | 30.68 | 0.23 |
|  | 39 | MOL002935 | Baicalin | 29.53 | 0.77 |
|  | 40 | MOL004684 | methyl (E)-octadec-2-enoate | 29.84 | 0.17 |
|  | 41 | MOL009730 | methyl icos-11-enoate | 29.49 | 0.23 |
|  | 42 | MOL012240 | 2',3',5,7-tetrahydroxyflavone | 25.75 | 0.24 |
|  | 43 | MOL000008 | apigenin | 23.06 | 0.21 |
|  | 44 | MOL002560 | chrysin | 22.61 | 0.18 |
|  | 45 | MOL002912 | Dihydrobaicalin | 20.85 | 0.75 |
|  | 46 | MOL000357 | Sitogluside | 20.63 | 0.62 |
|  | 47 | MOL012267 | Scutevulin | 20.67 | 0.27 |
| ZZ | 1 | MOL004561 | Sudan III | 84.07 | 0.59 |
|  | 2 | MOL007245 | 3-Methylkempferol | 60.16 | 0.26 |
|  | 3 | MOL003095 | 5-hydroxy-7-methoxy-2-(3,4,5-trimethoxyphenyl)chromone | 51.96 | 0.41 |
|  | 4 | MOL000098 | Quercetin | 46.43 | 0.28 |
|  | 5 | MOL009038 | GBGB | 45.58 | 0.83 |
|  | 6 | MOL001942 | Isoimperatorin | 45.46 | 0.23 |
|  | 7 | MOL000449 | Stigmasterol | 43.83 | 0.76 |
|  | 8 | MOL001494 | Mandenol | 42.00 | 0.19 |
|  | 9 | MOL000422 | kaempferol | 41.88 | 0.24 |
|  | 10 | MOL001641 | METHYL LINOLEATE | 41.93 | 0.17 |
|  | 11 | MOL000358 | beta-sitosterol | 36.91 | 0.75 |
|  | 12 | MOL001406 | crocetin | 35.30 | 0.26 |
|  | 13 | MOL001941 | Ammidin | 34.55 | 0.22 |
|  | 14 | MOL001506 | Supraene | 33.55 | 0.42 |
|  | 15 | MOL001663 | (4aS,6aR,6aS,6bR,8aR,10R,12aR,14bS)-10-hydroxy-2,2,6a,6b,9,9,12a-heptamethyl-1,3,4,5,6,6a,7,8,8a,10,11,12,13,14b-tetradecahydropicene-4a-carboxylic acid | 32.03 | 0.76 |
|  | 16 | MOL002883 | Ethyl oleate (NF) | 32.4 | 0.19 |
|  | 17 | MOL001745 | Methyl vaccenate | 31.90 | 0.17 |
|  | 18 | MOL002203 | Exceparl M-OL | 31.90 | 0.16 |
|  | 19 | MOL000263 | oleanolic acid | 29.02 | 0.76 |
|  | 20 | MOL003515 | (3S,4S,4aR,6aR,6bS,8aS,12aS,14aR,14bR)-3-hydroxy-4,6a,6b,11,11,14b-hexamethyl-1,2,3,4a,5,6,7,8,9,10,12,12a,14,14a-tetradecahydropicene-4,8a-dicarboxylic acid | 27.21 | 0.72 |
|  | 21 | MOL001652 | 1H-2,6-dioxacyclopent(cd)inden-1-one, 4-((acetyloxy)methyl)-5-(beta-D-glucopyranosyloxy)-2a,4a,5,7b-tetrahydro-, (2aS-(2aalpha,5alpha,7balpha))- | 26.43 | 0.71 |
|  | 22 | MOL001400 | 4,8,12,16-tetramethylheptadecan-4-olide | 26.06 | 0.20 |
|  | 23 | MOL007994 | ilexoside A_qt | 22.43 | 0.74 |
|  | 24 | MOL000551 | Hederagenol | 22.42 | 0.74 |
|  | 25 | MOL013377 | Lutein | 22.59 | 0.55 |
|  | 26 | MOL002560 | chrysin | 22.61 | 0.18 |
| WLX | 1 | MOL000449 | Stigmasterol | 43.83 | 0.76 |
|  | 2 | MOL005603 | Heptyl phthalate | 42.26 | 0.31 |
|  | 3 | MOL001641 | METHYL LINOLEATE | 41.93 | 0.17 |
|  | 4 | MOL005594 | ClematosideA'_qt | 37.51 | 0.76 |
|  | 5 | MOL005235 | Embelin | 37.72 | 0.18 |
|  | 6 | MOL000358 | Beta-sitosterol | 36.91 | 0.75 |
|  | 7 | MOL005598 | Embinin | 33.91 | 0.73 |
|  | 8 | MOL002372 | (6Z,10E,14E,18E)-2,6,10,15,19,23-hexamethyltetracosa-2,6,10,14,18,22-hexaene | 33.55 | 0.42 |
|  | 9 | MOL001663 | (4aS,6aR,6aS,6bR,8aR,10R,12aR,14bS)-10-hydroxy-2,2,6a,6b,9,9,12a-heptamethyl-1,3,4,5,6,6a,7,8,8a,10,11,12,13,14b-tetradecahydropicene-4a-carboxylic acid | 32.03 | 0.76 |
|  | 10 | MOL000508 | Friedelin | 29.16 | 0.76 |
|  | 11 | MOL000263 | oleanolic acid | 29.02 | 0.76 |
|  | 12 | MOL000551 | Hederagenol | 22.42 | 0.74 |
| TR | 1 | MOL001371 | Populoside_qt | 108.89 | 0.20 |
|  | 2 | MOL001351 | Gibberellin A44 | 101.61 | 0.54 |
|  | 3 | MOL001348 | Gibberellin 17 | 94.64 | 0.49 |
|  | 4 | MOL001353 | GA60 | 93.17 | 0.53 |
|  | 5 | MOL001349 | 4a-formyl-7alpha-hydroxy-1-methyl-8-methylidene-4aalpha,4bbeta-gibbane-1alpha,10beta-dicarboxylic acid | 88.6 | 0.46 |
|  | 6 | MOL001344 | GA122-isolactone | 88.11 | 0.54 |
|  | 7 | MOL001329 | 2,3-didehydro GA77 | 88.08 | 0.53 |
|  | 8 | MOL001360 | GA77 | 87.89 | 0.53 |
|  | 9 | MOL001340 | GA120 | 84.85 | 0.45 |
|  | 10 | MOL001339 | GA119 | 76.36 | 0.49 |
|  | 11 | MOL001358 | gibberellin 7 | 73.80 | 0.50 |
|  | 12 | MOL001342 | GA121-isolactone | 72.70 | 0.54 |
|  | 13 | MOL001361 | GA87 | 68.85 | 0.57 |
|  | 14 | MOL001355 | GA63 | 65.54 | 0.54 |
|  | 15 | MOL001343 | GA122 | 64.79 | 0.50 |
|  | 16 | MOL001352 | GA54 | 64.21 | 0.53 |
|  | 17 | MOL001328 | 2,3-didehydro GA70 | 63.29 | 0.50 |
|  | 18 | MOL001350 | GA30 | 61.72 | 0.54 |
|  | 19 | MOL001323 | Sitosterol alpha1 | 43.28 | 0.78 |
|  | 20 | MOL000493 | campesterol | 37.58 | 0.71 |
|  | 21 | MOL001368 | 3-O-p-coumaroylquinic acid | 37.63 | 0.29 |
|  | 22 | MOL000296 | hederagenin | 36.91 | 0.75 |
|  | 23 | MOL000358 | beta-sitosterol | 36.91 | 0.75 |
|  | 24 | MOL001324 | campesterol-3-O-β-D-(6-O-oleyl)glucopyranoside | 27.03 | 0.17 |
|  | 25 | MOL001317 | β-sitosterol 3-O-β-D-(6-O-oleyl)glucopyranoside | 26.94 | 0.16 |
|  | 26 | MOL001318 | β-sitosterol-3-(6-palmitoyl)glucopyranoside | 26.07 | 0.18 |
|  | 27 | MOL001325 | campesterol-3-O-β-D-(6-O-palmityl)glucopyranoside | 25.65 | 0.19 |
|  | 28 | MOL000295 | alexandrin | 20.63 | 0.63 |
|  | 29 | MOL001315 | campesterol-3-O-β-D-glucopyranoside | 20.49 | 0.67 |
|  | 30 | MOL001362 | GA95 | 20.01 | 0.49 |
| YYR | 1 | MOL000449 | Stigmasterol | 43.83 | 0.76 |
|  | 2 | MOL001323 | Sitosterol alpha1 | 43.28 | 0.78 |
|  | 3 | MOL001494 | Mandenol | 42.00 | 0.19 |
|  | 4 | MOL001641 | METHYL LINOLEATE | 41.93 | 0.17 |
|  | 5 | MOL000953 | CLR | 37.87 | 0.68 |
|  | 6 | MOL000359 | Sitosterol | 36.91 | 0.75 |
|  | 7 | MOL008121 | 2-Monoolein | 34.23 | 0.29 |
|  | 8 | MOL002882 | [(2R)-2,3-dihydroxypropyl] (Z)-octadec-9-enoate | 34.13 | 0.30 |
|  | 9 | MOL002372 | (6Z,10E,14E,18E)-2,6,10,15,19,23-hexamethyltetracosa-2,6,10,14,18,22-hexaene | 33.55 | 0.42 |
|  | 10 | MOL008118 | Coixenolide | 32.4 | 0.43 |
|  | 11 | MOL002875 | Methyl oleate | 31.90 | 0.17 |
|  | 12 | MOL000508 | Friedelin | 29.16 | 0.76 |
|  | 13 | MOL001884 | Omaine | 26.60 | 0.51 |

HQC, Huangqin Qingrechubi Capsule; TCM, traditional Chinese medicine; OB, oral bioavailability; DL, drug-likeness; HQ, Huangqin; ZZ, Zhizi; YYR, Yiyiren; TR, Taoren; WLX, Weilingxian.
