# Supplementary material for: The IL-17 pathway mediated by m6A-modified lncRNA H19: a new mechanism for Jianpi Qingre Tongluo Prescription in repressing inflammation and improving lipid metabolism in gout arthritis
Source: Chin Med. 2026 Mar 18;21:95. doi: 10.1186/s13020-026-01379-z (PMC12997696; doi:10.1186/s13020-026-01379-z)
Supplement: Supplementary file 9 — Additional file 9. [file 13020_2026_1379_MOESM9_ESM.docx]

**Supplementary table 4** MS data and identification results of chemical constituents of HQC

| No. | t_R_/min | Identified component | Negative ion (m/z) | | Positive ion (m/z) | | Formula | Adducts | MS/MS (m/z) | Source |
| --- | --- | --- | --- | --- | --- | --- | --- | --- | --- | --- |
|  |  |  | Indicated | ppm | Indicated | ppm |  |  |  |  |
| 1 | 1.67 | L-Glutamic acid | - | - | 148.0598 | -1.4 | C_5_H_9_NO_4_ | [M+H]^+^ | 134.0309, 131. 0530, 130.0499, 102. 0585, 85.0267 | TR |
| 2 | 1.70 | L-Histidine | - | - | 156.0766 | 8.8 | C_6_H_9_N_3_O_2_ | [M+H]^+^ | 112.0867, 110.0722, 101.0438, 83.0606 | TR |
| 3 | 1.72 | Cystine | - | - | 241.0345 | 7.9 | C_6_H_12_N_2_O_4_S_2_ | [M+H]^+^ | 190.9833, 163.0611,  147.0034 | TR |
| 4 | 1.73 | Adenosine | - | - | 268.1062 | 4.5 | C_10_H_13_N_5_O_4_ | [M+H]^+^ | 137.0639, 136.0619, 133.0495, 119.0377, 115.0395, 97.0279, 69.0326 | TR |
| 5 | 1.76 | 4-Hydroxycinnamic acid | - | - | 165.0558 | 3.9 | C_9_H_8_O_3_ | [M+H]^+^ | 147.0431, 130.0662, 123.0406, 106.9959 | YYR |
| 6 | 2.15 | D-tert-Leucine | - | - | 132.1023 | 2.2 | C_6_H_13_NO_2_ | [M+H]^+^ | 87.0406, 86.0981, 71.0121, 68.0229 | YYR |
| 7 | 2.60 | 4-Hydroxybenzoic acid | - | - | 161.0187 | -8.2 | C_7_H_6_O_3_ | [M+Na]^+^ | 121.0286, 99.5559, 79.0554, 77.0384 | YYR |
| 8 | 2.67 | Mandelicacid-β-Dgluco-pyranoside | 359.0989 | 5.3 | - | - | C_14_H_18_O_8_ | [M+HCOO]^-^ | 295.0832, 223.0614, 193.0591 | TR |
| 9 | 2.84 | Shanzhiside | 391.1256 | 2.84 | - | - | C_16_H_24_O_11_ | [M-H]^-^ | 229.0715, 185.0793 | ZZ |
| 10 | 2.97 | Quercetin-7-O-β-Dglucopyranoside | - | - | 487.0819 | -6.6 | C_21_H_20_O_12_ | [M+Na]^+^ | 305.0588, 145.0498, 99.0420, 97.0279 | TR |
| 11 | 2.98 | Geniposidic acid | 373.1154 | 3.1 | - | - | C_16_H_22_O_10_ | [M-H]^-^ | 211.0638, 167.0713, 149.0618, 143.0371, 123.0463 | ZZ |
| 12 | 3.53 | Protocatechuic acid | 153.0215 | 8.1 | - | - | C_7_H_6_O_4_ | [M-H]^-^ | 108.0268, 109.0220 | WLX |
| 13 | 3.97 | L-Tryptophan | 203.0835 | 4.3 | - | - | C_11_H_12_N_2_O_2_ | [M-H]^-^ | 186.0574, 159.0959, 142.0674, 130.0695, 116.0526 | TR |
| 14 | 4.47 | Chlorogenic acid | 353.0883 | 4.0 | - | - | C_16_H_18_O_9_ | [M-H]^-^ | 354.0962, 352.0781, 192.0542, 191.0547 | TR |
| 15 | 4.53 | Genipin-1-β-Dgentiobioside | 595.1836 | -3.0 | 573.1764 | -1.4 | C_23_H_34_O_15_ | [M+HCOO]^-^  [M-H]^-^  [M+Na]^+^  [M+H]^+^ | 549.1795, 225.0764, 207.0647, 551.1968, 227.0911, 209.0829,  177.0554 | ZZ |
| 16 | 4.66 | Caffeic acid | - | - | 181. 047 9 | 6.3 | C_9_H_8_O_4_ | [M+H]^+^  [M+Na]^+^ | 179.0324, 139.0356, 135.0445, 109.0291 | YYR |
| 17 | 4.94 | Methyl-7-ethoxy-3-indolecarbonate | - | - | 220.0960 | 2.4 | C_12_H_13_NO_3_ | [M+H]^+^ | 178.1217, 162.0748 | WLX |
| 18 | 4.94 | Amygdalin | 502.1563 | -3.4 | 480.1469 | -3.0 | C_20_H_27_NO_11_ | [M+HCOO]^-^  [M-H]^-^  [M+Na]^+^  [M+H]^+^ | 457.1543, 456.1488, 324.1019, 179.9435, 113.0269/458.1670,  297.1190, 296.1145, 197.0835, 163.0611 | TR |
| 19 | 5.16 | Myristicin | - | - | 193.0876 | 8.3 | C_11_H_12_O_3_ | [M+H]^+^ | 152.0430, 105.0698, 79.0572, 77.0378 | WLX |
| 20 | 5.29 | Cinnamic acid | - | - | 149.0600 | -3.5 | C_9_H_8_O_2_ | [M+H]^+^ | 131.0483, 107.0488, 104.0570, 103.0532, 77.0378 | WLX |
| 21 | 5.29 | Genipin | 225.0764 | 2.1 | 227.0911 | 2.6 | C_11_H_14_O_5_ | [M-H]^-^  [M+H]^+^ | 207.0647, 177.0554, 131.0483, 121.0647, 103.0532 | ZZ |
| 22 | 5.30 | Geniposide | 433.1325 | -3.6 | 411.1251 | -3.2 | C_17_H_24_O_10_ | [M+HCOO]^-^  [M-H]^-^  [M+H]^+^  [M+Na]^+^ | 387.1265, 179.0536, 119.0357，249.0751, 231.0618, 217.0463,  203. 0520, 185.0403, 147.0431 | ZZ |
| 23 | 5.31 | Formononetol | - | - | 291.0687 | 0.1 | C_16_H_12_O_4_ | [M+Na]^+^ | 269.0889, 254.0509, 226.0622 | WLX |
| 24 | 5.96 | Prunasin | 340.1017 | 1.0 | - | - | C_14_H_17_NO_6_ | [M+HCOO]^-^  [M-H]^-^ | 218.0606, 223.0614, 234.0680, 161.0449 | TR |
| 25 | 6.30 | Benzyl-β-D-glucopyrano-β-D-side | 269.0973 | -4.4 | 271.0596 | -4.2 | C_13_H_18_O_6_ | [M-H]^-^  [M+H]^+^ | 139.0766, 161.0241 | TR |
| 26 | 6.47 | 2-Hydroxyphenylacetic acid | - | - | 153.0541 | 8.9 | C_8_H_8_O_3_ | [M+H]^+^ | 135.0445, 108.0548, 107.0488，79.0536, 77.0396 | YYR |
| 27 | 6.50 | Rutin | 609.1441 | 0.0 | 633.1476 | 4.8 | C_27_H_30_O_16_ | [M-H]^-^  [M+H]^+^  [M+Na]^+^ | 449.1111, 301.0379, 611.1635, 303.0533 | ZZ |
| 28 | 6.67 | Chrysin-6-C-β-Dglucoside-8-C-α-Larabinoside | 547.1439 | -3.5 | - | - | C_26_H_28_O_13_ | [M-H]^-^ | 457.1149, 367.0822, 337.0710 | HQ |
| 29 | 6.67 | Chrysin-6-C-β-Dglucoside-8-C-β-Larabinoside | - | - | 571.1401 | -2.2 | C_26_H_28_O_13_ | [M+Na]^+^  [M+H]^+^ | 549.1616, 363.0853, 333.0785, 309.0788 | HQ |
| 30 | 6.92 | 3-O(-E)-Feruloylquinic acid | - | - | 369.1192 | 6.2 | C_17_H_20_O_9_ | [M+H]^+^ | 189.0932, 177.0554, 146.0324，117.0323 | TR |
| 31 | 6.94 | Acteoside | 623.1949 | -2.2 | - | - | C_29_H_36_O_15_ | [M-H]^-^ | 364.0821, 311.0944, 161.0241, 133.0282 | HQ |
| 32 | 6.94 | Scutellarin | 461.0774 | -0.3 | 485.0696 | 6.7 | C_21_H_18_O_12_ | [M-H]^-^  [M+H]^+^  [M+Na]^+^ | 285.0833, 113.0269, 463, 0950, 269.0486 | HQ |
| 33 | 6.96 | Sinapic acid | 223.0614 | 5.3 | - | - | C_11_H_12_O_5_ | [M-H]^-^ | 208.0373, 193.0137, 179.0700, 165.0522, 149.0268 | ZZ |
| 34 | 6.97 | Scutellarein | 285.0388 | 3.9 | - | - | C_15_H_10_O_6_ | [M-H]^-^ | 255.0271, 239.0392, 139.0045, 136.9882, 119.0535, 117.0332 | HQ |
| 35 | 7.13 | Quercetin | 301.0343 | 3.9 | 303.0533 | 6.9 | C_15_H_10_O_7_ | [M-H]^-^  [M+H]^+^ | 161.0241, 151.0038, 149.0268, 121.0259, 286.0524, 285.0427, 275.0567, 258.0525 | TR |
| 36 | 7.13 | 6′-O-trans-Sinapoyl gardoside | 579.1722 | -0.6 | - | - | C_27_H_32_O_14_ | [M-H]^-^ | 223.0614, 193.0478, 167.0713, 123.0463 | ZZ |
| 37 | 7.34 | Ferulic acid | - | - | 195.0661 | 6.5 | C_10_H_10_O_4_ | [M+H]^+^ | 177.0554, 149.0600, 145.0300 | WLX |
| 38 | 7.35 | 2′-O-cis-Coumaroyl  gardoside | 519.1495 | 1.5 | - | - | C_25_H_28_O_12_ | [M-H]^-^ | 307.0807, 211.0638, 149.0618, 145.0282 | ZZ |
| 39 | 7.41 | Nobiletin | - | - | 403.1351 | 6.1 | C_21_H_22_O_8_ | [M-H]^-^ | 373.0812, 241.0504, 105.0558 | WLX |
| 40 | 7.44 | Kaempferol-3-rutinoside | 593.1541 | 1.0 | 595.1712 | -4.8 | C_27_H_30_O_15_ | [M-H]^-^  [M+H]^+^ | 284.0312, 227.0326, 329.0794, 288.0600, 287.0571, 145.0498 | TR |
| 41 | 7.50 | Naringin | - | - | 603.1710 | 3.5 | C_27_H_32_O_14_ | [M+Na]^+^ | 384.1187, 315.0836, 309.1111, 273.0714, 153.0186 | TR |
| 42 | 7.87 | Crocin I | 10213.376 | -3.1 | - | - | C_44_H_64_O_24_ | [M+HCOO]^-^  [M-H]^-^ | 975.3691, 651.2560, 327.1585 | ZZ |
| 43 | 8.00 | Hesperetin | - | - | 303.0890 | 6.7 | C_16_H_14_O_6_ | [M+H]^+^ | 287.0571, 270.0565, 219.0649, 201.0536, 178.0589, 153.0186, 146.0324 | WLX |
| 44 | 8.01 | 4″-O-trans-P-Coumaroyl genipin gentiobioside | 695.2158 | -3.1 | - | - | C_32_H_40_O_17_ | [M-H]^-^ | 469.1335, 225.0764, 163.0397, 123.0440 | ZZ |
| 45 | 8.01 | 5-O-Feruloyl-3-O-(β-D-glucopyranosyl)-2-deoxy-D-ribono-γ-lac⁃tone | - | - | 471.1492 | 0.7 | C_21_H_26_O_12_ | [M+H]^+^  [M+Na]^+^ | 294.2265 | WLX |
| 46 | 8.01 | (-)-Catechin | - | - | 291.0897 | 5.2 | C_15_H_14_O_6_ | [M+H]^+^ | 290.0432, 273.0951, 207.0697, 177.0554, 147.0431 | TR |
| 47 | 8.11 | 6″-O-trans-Sinapoyil  genipin gentiobioside | 755.2365 | -2.1 | - | - | C_34_H_44_O_19_ | [M-H]^-^ | 529.1582, 225.0764 | ZZ |
| 48 | 8.26 | Azelaic acid | 187.0969 | 0.2 | - | - | C_9_H_16_O_4_ | [M-H]^-^ | 174.0943, 135.0826, 103.0554, 93.0698 | YYR |
| 49 | 8.57 | Luteolin | 285.0388 | 1.4 | - | - | C_15_H_10_O_6_ | [M-H]^-^ | 257.0435, 213.0568, 211.0341, 133.0282 | WLX |
| 50 | 8.57 | Kaempferol | - | - | 287.0525 | 3.0 | C_15_H_10_O_6_ | [M+H]^+^ | 240.0429, 188.0495, 164.0469, 93.3925 | TR |
| 51 | 8.65 | 6′-O-trans-Sinapoyl  jasminoside B | 551.1839 | -1.0 | - | - | C_27_H_36_O_12_ | [M-H]^-^ | 385.1165, 223.0614 | ZZ |
| 52 | 8.71 | Crocin Ⅱ | 859.3249 | -0.4 | - | - | C_38_H_54_O_19_ | [M+HCOO]^-^ | 651.2656, 327.1585, 179.0591 | ZZ |
| 53 | 8.92 | Lariciresinol | 359.1454 | 3.5 | - | - | C_20_H_24_O_6_ | [M+HCOO]^-^  [M-H]^-^ | 330.1440, 208.0757, 192.0797, 180.0624, 176.0714, 149.0493 | WLX |
| 54 | 9.06 | 6′-O-E-Sinapoyil geniposide | 593.1839 | 1.3 | - | - | C_28_H_34_O_14_ | [M-H]^-^ | 367.1018, 190.0269, 123.0463，101.0274 | ZZ |
| 55 | 9.06 | Baicalin | 445.0753 | -3.7 | - | - | C_21_H_18_O_11_ | [M-H]^-^ | 269.0436, 251.0343, 175.0251, 169.0657 | HQ |
| 56 | 9.06 | Glychionide A | - | - | 447.0911 | -6.2 | C_21_H_18_O_11_ | [M+H]^+^  [M+Na]^+^ | 271.0596 | HQ |
| 57 | 9.06 | Viscidulin Ⅲ | - | - | 347.0790 | 0.6 | C_17_H_14_O_8_ | [M+H]^+^ | 332.0522, 314.0419, 289.0368，169.0124 | HQ |
| 58 | 9.52 | Carthamidin | - | - | 289.0716 | 4.1 | C_15_H_12_O_6_ | [M+H]^+^ | 153.0186, 147.0431 | HQ |
| 59 | 9.52 | (±)-Dihydrokaempferol | 287.0560 | -0.5 | - | - | C_15_H_12_O_6_ | [M-H]^-^ | 270.0479, 269.0436, 245.0705, 175.0332, 111.0090 | TR |
| 60 | 9.64 | Prunin | - | - | 457.1163 | 0.4 | C_21_H_22_O_10_ | [M+Na]^+^ | 435.1255, 273.0748, 153.0186, 147.0431, 91.0547 | TR |
| 61 | 9.64 | (+/-)-Naringenin | 271.0607 | -0.2 | 273.0748 | -4.1 | C_15_H_12_O_5_ | [M-H]^-^  [M+H]^+^  [M+Na]^+^ | 125.0262, 255.0696, 237.0573, 232.0708, 231.0681, 189.0594，  107.0488 | TR, WLX |
| 62 | 9.77 | 5-Deoxykaempferol | 269.0436 | 9.78 | - | - | C_15_H_10_O_5_ | [M-H]^-^ | 240.0429, 239.0329, 224.0463, 197.0591, 121.0304 | WLX |
| 63 | 9.77 | Apigenin-7-O-β-D-glucuronide | 445.0753 | -4.7 | - | - | C_21_H_18_O_11_ | [M-H]^-^ | 891.1583, 269.0436 | HQ |
| 64 | 9.92 | Apigenin-7-O-β-D-glucoside | 431.0956 | 2.2 | - | - | C_21_H_20_O_10_ | [M-H]^-^ | 151.0038, 239.0329, 240.0429, 268.0379, 432.1028 | HQ |
| 65 | 10.26 | Chrysin-7-O-β-D-glucoronide | 429.0804 | -3.7 | - | - | C_21_H_18_O_10_ | [M-H]^-^ | 253.0479, 175.0251 | HQ |
| 66 | 10.26 | Daidzein | 253.0511 | 0.8 | - | - | C_15_H_10_O_4_ | [M+HCOO]^-^  [M-H]^-^ | 224.0494, 196.0581, 180.0596, 169.0604, 117.0309 | WLX |
| 67 | 10.84 | Oroxylin A 7-O-β-D-glucuronide | - | - | 461.1066 | -5.4 | C_22_H_20_O_11_ | [M+H]^+^  [M+Na]^+^ | 431.0976, 285.0772 | HQ |
| 68 | 11.19 | Genistein | - | - | 271.0596 | 3.3 | C_15_H_10_O_5_ | [M+H]^+^ | 243.0690, 153.0186, 149.0226 | WLX |
| 69 | 12.11 | Matairesinol | 357.1323 | 8.6 | - | - | C_20_H_22_O_6_ | [M-H]^-^ | 342.1193, 258.9164, 161.0683, 153.0620 | WLX |
| 70 | 13.17 | Apigenin | - | - | 271.0630 | 8.0 | C_15_H_10_O_5_ | [M+H]^+^ | 242.0618, 152.0632, 145.0498, 131.0834, 91.0552 | HQ |
| 71 | 13.25 | Crocin Ⅲ | 697.2724 | 3.0 | - | - | C_32_H_44_O_14_ | [M+HCOO]^-^ | 651.2656, 327.1585 | ZZ |
| 72 | 13.43 | 5, 7, 4'-Trihydroxy-  6-methoxyflavanone | 299.0570 | 4.0 | - | - | C_16_H_12_O_6_ | [M-H]^-^ | 284.0312 | HQ |
| 73 | 13.88 | Baicalein | 269.0470 | 1.6 | 271.0596 | -4.8 | C_15_H_10_O_5_ | [M-H]^-^ | 251.0375, 241.0519, 195.0454, 169.0657 | HQ |
| 74 | 16.50 | Diethyl phthalate | - | - | 245.0832 | 3.5 | C_12_H_14_O_4_ | [M+Na]^+^ | 177.0554, 150.0260, 149.0226, 121.0286 | WLX |
| 75 | 19.34 | Genkwanin | 283.0598 | -0.9 | - | - | C_16_H_12_O_5_ | [M-H]^-^ | 268.0379, 267.0272, 240.0429, 239.0329 | HQ |
| 76 | 19.51 | Chrysin | 253.0511 | 0.3 | 255.0631 | 4.7 | C_15_H_10_O_4_ | [M-H]^-^ | 143.0518 | HQ |
| 77 | 19.74 | Wogonin | 283.0632 | -0.1 | - | - | C_16_H_12_O_5_ | [M-H]^-^ | 268.0379, 240.0429, 239.0329, 224.0463 | HQ |
| 78 | 19.75 | Isoliquiritigenin | - | - | 257.0804 | 4.3 | C_15_H_12_O_4_ | [M+H]^+^ | 242.0586, 239.0718, 153.0693, 137.0232 | HQ |
| 79 | 19.75 | Liquiritigenin | 255.0695 | 3.8 | - | - | C_15_H_12_O_4_ | [M-H]^-^ | 135.0105, 119.0178 | WLX |
| 80 | 19.76 | 5, 2'-Dihydroxy-7, 8-dimethoxyflavone | 313.0713 | 0.5 | - | - | C_17_H_14_O_6_ | [M-H]^-^ | 298.0461, 283.0254, 164.9836 | HQ |
| 81 | 20.60 | 3-Oxo-oleanolic acid | 453.3350 | 3.34 | - | - | C_30_H_46_O_3_ | [M+HCOO]^-^  [M-H]^-^ | 453.3350 | WLX |
| 82 | 20.73 | Vitamin E | - | - | 453.3700 | 6.5 | C_29_H_50_O_2_ | [M+Na]^+^ | 205.1248, 191.1078, 177.0936, 165.0926 | TR |

HQC, Huangqin Qingrechubi Capsule; HQ, Huangqin; ZZ, Zhizi; YYR, Yiyiren; TR, Taoren; WLX, Weilingxian; MS, mass spectrometry.
