# Supplementary material for: The IL-17 pathway mediated by m6A-modified lncRNA H19: a new mechanism for Jianpi Qingre Tongluo Prescription in repressing inflammation and improving lipid metabolism in gout arthritis
Source: Chin Med. 2026 Mar 18;21:95. doi: 10.1186/s13020-026-01379-z (PMC12997696; doi:10.1186/s13020-026-01379-z)
Supplement: Supplementary file 10 — Additional file 10. [file 13020_2026_1379_MOESM10_ESM.docx]

**Supplementary table 5** The binding energies of active ingredients with ALKBH5 and FTO

| Protein (PDB ID) | Active ingredients | Binding energy (kcal/mol) |
| --- | --- | --- |
| ALKBH5 (4NRM) | MOL002910 (Carthamidin) | -7.11 |
| ALKBH5 (4NRM) | MOL002714 (Baicalein) | -5.44 |
| ALKBH5 (4NRM) | MOL002560 (Chrysin) | -6.55 |
| ALKBH5 (4NRM) | MOL000422 (Kaempferol) | -5.80 |
| ALKBH5 (4NRM) | MOL000173 (Wogonin) | -5.80 |
| ALKBH5 (4NRM) | MOL000098 (Quercetin) | -6.50 |
| ALKBH5 (4NRM) | MOL000008 (Apigenin) | -5.71 |
| ALKBH5 (4NRM) | MOL009038 (GBGB) | -4.24 |
| FTO (5ZMD) | MOL002910 (Carthamidin) | -6.12 |
| FTO (5ZMD) | MOL002714 (Baicalein) | -5.27 |
| FTO (5ZMD) | MOL002560 (Chrysin) | -5.75 |
| FTO (5ZMD) | MOL000422 (Kaempferol) | -5.76 |
| FTO (5ZMD) | MOL000173 (Wogonin) | -5.23 |
| FTO (5ZMD) | MOL000098 (Quercetin) | -4.33 |
| FTO (5ZMD) | MOL000008 (Apigenin) | -4.39 |
| FTO (5ZMD) | MOL009038 (GBGB) | -1.45 |

ALKBH5, AlkB homolog 5; FTO, fat mass and obesity-associated protein.
